# Tuning Single‐Molecule Conductance in Metalloporphyrin‐Based Wires via Supramolecular Interactions

**DOI:** 10.1002/anie.202007237

**Published:** 2020-08-24

**Authors:** Albert C. Aragonès, Alejandro Martín‐Rodríguez, Daniel Aravena, Josep Puigmartí‐Luis, David B. Amabilino, Núria Aliaga‐Alcalde, Arántzazu González‐Campo, Eliseo Ruiz, Ismael Díez‐Pérez

**Affiliations:** ^1^ Department of Chemistry Faculty of Natural & Mathematical Sciences King's College London Britannia House, 7 Trinity Street London SE1 1DB UK; ^2^ Departament de Química Inorgànica i Orgànica Diagonal 645 08028 Barcelona Spain; ^3^ Institut de Química Teòrica i Computacional Universitat de Barcelona Diagonal 645 08028 Barcelona Spain; ^4^ ICMAB-CSIC (Institut de Ciència dels Materials de Barcelona) Campus de la Universitat Autònoma de Barcelona 08193 Bellaterra Spain; ^5^ ICREA (Institució Catalana de Recerca i Estudis Avançats) Passeig Lluis Companys 23 08010 Barcelona Spain; ^6^ The GSK Carbon Neutral Laboratories for Sustainable Chemistry The University of Nottingham Triumph Road Nottingham NG7 2TU UK; ^7^ Institute of Chemical and Bioengineering ETH Zurich Vladimir Prelog Weg 1 8093 Zurich Switzerland; ^8^ Departamento de Química de los Materiales Facultad de Química y Biología Universidad de Santiago de Chile (USACH) Casilla 40, Correo 33 Santiago Chile; ^9^ Current address: Molecular Spectroscopy Department Max Planck Institute for Polymer Research Ackermannweg 10 55128 Mainz Germany

**Keywords:** biomolecular electronics, density functional calculations, metalloporphyrins, single-molecule junctions, supramolecular electronics

## Abstract

Nature has developed supramolecular constructs to deliver outstanding charge‐transport capabilities using metalloporphyrin‐based supramolecular arrays. Herein we incorporate simple, naturally inspired supramolecular interactions via the axial complexation of metalloporphyrins into the formation of a single‐molecule wire in a nanoscale gap. Small structural changes in the axial coordinating linkers result in dramatic changes in the transport properties of the metalloporphyrin‐based wire. The increased flexibility of a pyridine‐4‐yl‐methanethiol ligand due to an extra methyl group, as compared to a more rigid 4‐pyridinethiol linker, allows the pyridine‐4‐yl‐methanethiol ligand to adopt an unexpected highly conductive stacked structure between the two junction electrodes and the metalloporphyrin ring. DFT calculations reveal a molecular junction structure composed of a shifted stack of the two pyridinic linkers and the metalloporphyrin ring. In contrast, the more rigid 4‐mercaptopyridine ligand presents a more classical lifted octahedral coordination of the metalloporphyrin metal center, leading to a longer electron pathway of lower conductance. This works opens to supramolecular electronics, a concept already exploited in natural organisms.

## Graphical abstract

Supramolecular wires are created in a confined nanoscale junction by using metalloporphyrin coordination chemistry in a similar fashion to that found in bacteria nanowires. Slight chemical changes in the axial ligands and in the porphyrin ring determine the exact final supramolecular scaffold, which defines the electron pathway along the supramolecular wire.

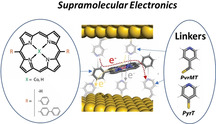

## Introduction

The concept of Supramolecular Electronics arises as a result of blending the studies of organic crystalline systems and the field of conducting polymers.^1^ In parallel, in the last decade, new crystal structure information on natural biomolecular wires has fascinated the scientific community by revealing the way nature exploits supramolecular electronics using arrays of axially coordinated metalloporphyrins to create highly efficient molecular conduits.^2^, ^3^ Supramolecular electronics also provides a unique opportunity to study the mechanobiology of electrical signaling, as another key aspects of the emerging field of mechanochemistry^4^ in biological systems.^5^ Metalloporphyrins have been extensively studied as molecular wires owing to a number of appealing properties such as high chemical stability and conjugation, modular metal center and rich supramolecular chemistry.^6^, ^7^, ^8^, ^9^, ^10^ Metalloporphyrins have been chemically connected to metal electrodes either by directly lying flat on the metal surfaces via π‐orbital interactions between the metal and the porphyrin ring,^11^, ^12^ or by covalent electrode/molecule attachment through porphyrin ring substituents.^13^, ^14^, ^15^, ^16^, ^17^, ^18^ Although the latter results in a robust, straightforward method to wire oligo‐porphyrins between two electrodes, the inclusion of such anchoring Scheme precludes the exploitation of other sources of supramolecular interactions that might lead to the formation of more efficient electron pathways already exploited in the natural biomolecular homologous wire. We have recently reported a novel way to form highly conductive metalloporphyrin wires by coordinating axial positions of the metalloporphyrin ring allowing to orient the ring plane perpendicularly to the electron pathway (main junction axis).^19^, ^20^, ^21^ This is possible thanks to the highly axial coordinative affinity of both metal center and porphyrin ring to strong Lewis bases. Such axial ligands act as anchoring groups or *linkers*,^22^ mimicking the common natural schemes exploited in the chemistry of photosynthetic and transmembrane electron transport.^2^, ^3^, ^23^

**Figure 1:**
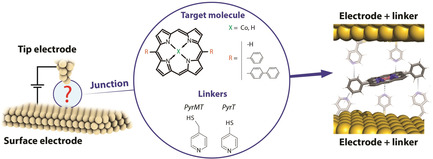
Schematic representation of the supramolecular architecture used to form metalloporphyrin molecular junctions in a STM tunneling gap using pyridine‐4‐yl‐methanethiol (*PyrMT*) and 4‐pyridinethiol (*PyrT*) linkers.

In this contribution, we aim to rationalize the conductance landscape of a metalloporphyrin‐based supramolecular wire under mechanical stress by systematically introducing structural changes of both the axial coordinative ligands and the porphyrin chemical substitution. To this goal, we built single‐molecule junctions using an STM‐break junction approach of Co^II^‐porphyrins (Figure 1) with different phenyl substitutions; unsubstituted (*P*), 5,15‐diphenyl (*DDP*) and 5,15‐dibisphenyl (*DBP*) substituted metalloporphyrins, employing thiol‐functionalized electrodes with two different pyridine compounds as axial coordinative linkers; a pyridine‐4‐yl‐methanethiol (*PyrMT*)^19^, ^20^, ^21^ and a 4‐pyridinethiol (*PyrT*). We show that this slight structural change in the axial ligands (differing by one methyl group) results in pronounced changes in the dominant supramolecular interactions that lead to the final molecular wire geometry, and ultimately dictates its final transport properties. We perform extensive DFT structural and charge transport simulations of the Co‐porphyrin supramolecular wire using the two axial ligands to help visualizing the most plausible junction configurations in each of the studied cases.

## Results and Discussion

### Single‐molecule charge transport of the *Co‐DPP/PyrMT* and *Co‐DPP/PyrT* assemblies

**Figure 2:**
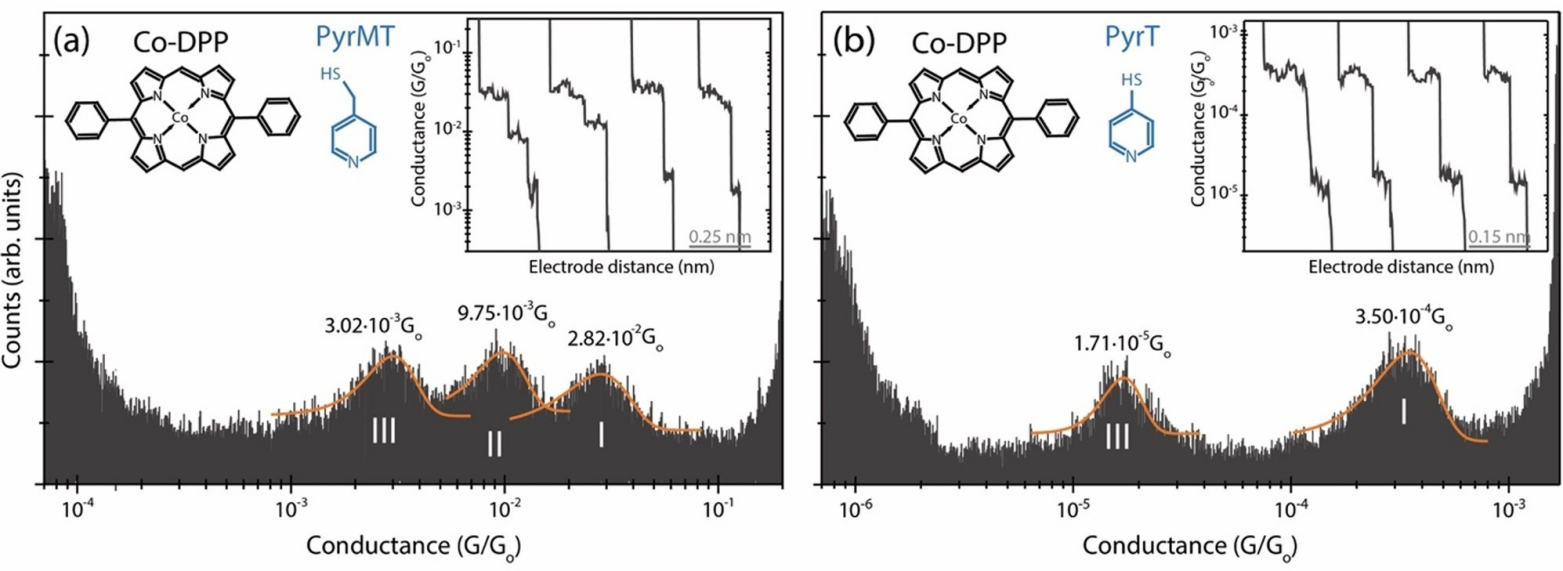
1D semi‐log conductance histograms of the *Co‐DPP* junctions using *PyrMT* (a) and *PyrT* (b) functionalized junction electrodes. The most probable conductance values are extracted from Gaussian fits of the peaks. The insets show representative individual current traces used to build the 1D histograms (see corresponding 2D histograms in Supporting Information (SI) Section S2). Feature I shows strong correlation (consecutive appearance) with both features II and III (see extended discussion on junction dynamics in SI Section S2 and S3). Counts have been normalized versus the total amount of counts. The applied bias voltage was set to +7.5 mV.

The STM‐BJ technique^24^ was used to form and measure the conductance of individual *Co‐DPP* dissolved in a non‐polar organic medium when they get trapped between the two Au electrodes of a STM junction functionalized with either *PyrMT* or *PyrT* linkers. The electrode functionalization is done ex‐situ by exposing the electrodes to a solution of each thiolated linker (see details in Supplementary Information (SI) section 1 and 6). We employed the dynamic STM‐BJ method, referred as *tapping*,^24^ where the STM tip electrode is driven repeatedly in and out of contact with the substrate electrode (see details in SI section 6). During the retraction cycle, individual *Co‐DPP* dissolved in the working media can spontaneously span the electrodes gap forming a transient molecular junction. Characteristic *plateaus*‐like features show up in the current versus separation (retraction) curves as a result of molecular wire formation and breakdown (see representative ones in Figure 2 insets). Typically, several hundred (up to a thousand) of those retraction curves displaying plateaus features are selected and accumulated in 1D and 2D semi‐log conductance histograms (details in SI section 2 and 6) resulting in prominent peaks, which provide the most probable single‐molecule conductance values. Figure 2 a and b show the corresponding 1D histograms for *Co‐DPP* junctions employing the *PyrMT* and *PyrT* linkers, respectively (see corresponding 2D histograms in SI section 2). Both junctions present multiple conductance features (peaks I to III) that are attributed to stable pyridine/metalloporphyrin interactions as the STM gap expands giving rise to different supramolecular associations. Experiments in the absence of either *Co‐DPP* (*PyrMT* and *PyrT* only) or linkers (*Co‐DPP* only) show no evident peak features within the same conductance range (in SI section 4.2). The *DPP* (metal‐free metalloporphyrin) junctions in the presence of both *PyrMT* and *PyrT* linkers show both the absence of the highest peak I conductance signature (see SI Section 4.1), which reveals that the peak I feature results from the axial coordination to the metal center in both cases, while peaks II and III are the result of pyridine/porphyrin ring interactions.^19^ The final junction's geometries are, however, unknown. The multiple conductance features observed in all the above studied cases bring several findings: (i) the more flexible *PyrMT* linker allows a larger number of possible stable junction's geometries, (ii) the geometries achieved with the flexible *PyrMT* linker are two orders of magnitude more conductive than those with the more rigid *PyrT*, and (iii) two well differentiated interactions, via metal or via porphyrin ring, take place with both linkers.

### Computational and ellipsometry analysis of the high conductance feature I in the *Co‐DPP/PyrMT* and *Co‐DPP/PyrT* assemblies

**Figure 3:**
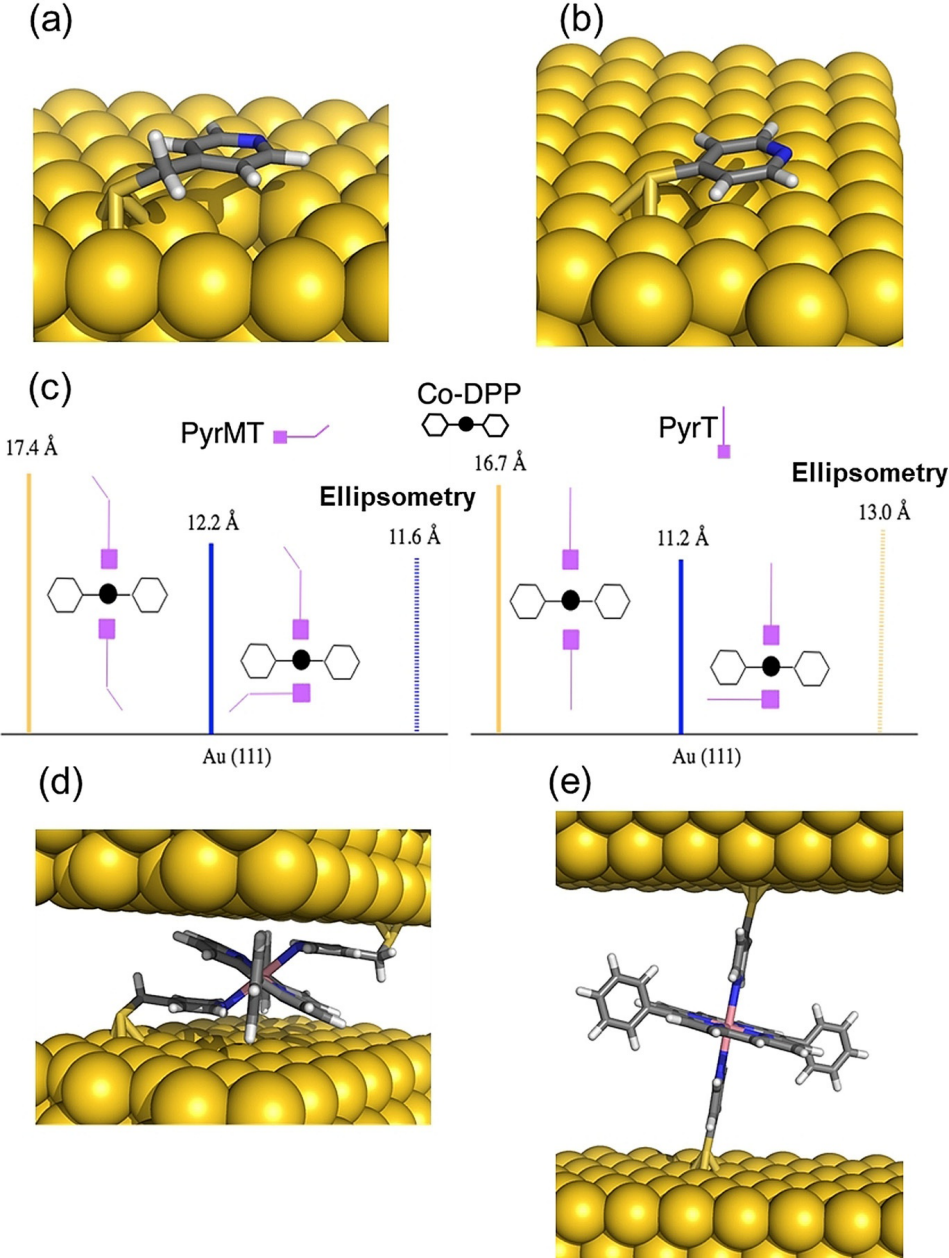
DFT‐optimized structures for the *PyrMT* (a) and *PyrT* (b) ligands adsorbed on Au(111). c) Schematic representation of both lying down and lifted *Co‐DPP*/linkers (*PyrMT* and *PyrT*) geometries, together with thicknesses determined from theoretical cases (solid lines) and ellipsometry values (stripped lines). Optimized full junction structures ascribed to the highest conductance signatures labelled as I in Figure 2 a, b, for the *PyrMT* (d) and *PyrT* (e) *Co‐DPP*/linker systems.

Conductance feature I results from the most stable (see SI section 3) metal‐mediated supramolecular interaction. We have performed density functional theory (DFT) calculations (see details in the Experimental Section and SI section 5) to identify the dominant chemical interactions in the linker/*Co‐DPP*/linker junction for the two studied linkers, and deduce plausible geometries related to the observed conductance signature I (Figure 2 a,b). We had initially proposed an axial coordination Scheme for the pyridine linkers standing perpendicular to the porphyrin plane^19^, ^21^ (Figure 1 right), inspired by the crystal structure of similar metal complexes.^25^ However, the computed conductance resulting from the transmission function using PBE+U functional for a *Co‐DPP* coordinated by two “standing up” *PyrMT* linkers results in a value of ≈10^−5^ G_0_ (see SI Figure S5.9), which is three orders of magnitude below the experimental value (2.82×10^−2^ G_o_). Such discrepancy is too large to be associated with DFT approximations, including PBE+U functional corrections for the well‐known underestimated highest (lowest) occupied (unoccupied) molecular orbitals (HOMO–LUMO) energy gap in the GGA functionals, which usually results in even larger (overestimated) conductance values. We then performed a detailed structural analysis of the most likely geometries of the linker/*Co‐DPP*/linker adduct in a constrained tunneling gap. The geometry of the *PyrMT* and *PyrT* linkers alone on the electrode surface was optimized using a many‐body approach to include the dispersion term.^26^, ^27^ The *PyrMT* shows a much larger propensity to “lie down” on the electrode surface (Figure 3 a), scoring 14.0 kcal mol^−1^ more stable than a “standing up or tilted” geometry. The *PyrT* linker, on the other hand, computes a much lower 6.0 kcal mol^−1^ difference between both conformations, adopting a slightly higher tilt angle (Figure 3 b), and suggesting higher likeliness of finding the linker in a “lifted” geometry when forming part of a compact monolayer, as the ones prepared in the experiments (see SI section 6). Moreover, a standing up geometry of an adsorbed *PyrT* in a compact monolayer on gold has been previously suggested from STM imaging.^28^, ^29^ The above computational results (Figure 3 a,b) suggest the *PyrMT* and *PyrT* might interact with the *Co‐DPP* in a π‐π stacking and in a more axial coordination, respectively. To corroborate this scenario, we have also performed ellipsometry measurements on a molecular layer of the form linker*/Co‐DPP/*linker for both *PyrMT* and *PyrT* linkers on a clean Au surface (Figure 3 c, Experimental Section and SI Section 1). The resulting thickness values are consistent with the formation of a Au/*PyrMT*(lying down)/*Co‐DPP/PyrMT*, scoring the lowest measured thickness (11.6 Å), and a thicker (13.0 Å) Au/*PyrT*(lifted)/*Co‐DPP/PyrT*, thus supporting our initial hypothesis: in the presence of *Co‐DPP*, the adsorbed *PyrMT* and *PyrT* linkers on the Au surface adopt a lying down and lifted geometries, respectively. Note that the ellipsometry analysis corresponds to the structure of the linker monolayer only (Figure 3 c) and that the final geometry upon molecular junction formation (two electrodes) might differ from the one in the ellipsometry experiment (one electrode). Notwithstanding, the ellipsometry data highlights a completely distinct pyridine interaction to the metal electrodes, resulting in the more rigid *PyrT* linker significantly decoupled from the metal surface (thicker measured layer) in comparison with the more flexible *PyrMT* (thinner measured layer), despite the larger molecular length of the latter.

DFT‐optimized structures for the whole linker/*Co‐DPP*/linker supramolecular junction for the two linkers are shown in Figure 3 d and e, and the computed conductance values from the corresponding zero‐energy transmission functions are 6.84×10^−2^ G_0_ and 2.46×10^−4^ G_0_ for the *PyrMT* and *PyrT* junctions, respectively, in excellent agreement with the experimental data corresponding to the peak I features, 2.82×10^−2^ G_0_ and 3.50×10^−4^ G_0_, respectively (Figure 2). The major supramolecular interactions lead in each case to a completely different junction; the *PyrMT* linker forms a π‐stacked conformation coordinating the metal center, while the *PyrT* preferably coordinates the metal center in a quasi‐fully standing up fashion. Figure 3 d and e configurations are then ascribed to the most likely molecular wire configurations leading to transmissions given by the peaks I in Figure 2 a and b. Note that the experimental (dynamic) tilted angle of the *PyrT* might differ from the suggested quasi‐fully standing up conformation in Figure 3 e, as deduced from the ellipsometry data. With independence of the final exact tilted angle in the molecular junction, a major impact on conductance due to the *PyrT*‐pyridine decoupling from the Au surface is observed. We conclude here that the larger flexibility of the *PyrMT* promotes the stabilization of the final junction structure through π‐π stacking interactions, while the rigid *PyrT* tends to adopt a more orthogonal coordinative geometry. The shorter conduction path in the *PyrMT* junction justifies its larger transmission. It is also important to remark the unusual coordination geometry of the metalloporphyrin with the *PyrMT* ligand, which evidences the interplay between linker‐linker neighbor interactions and linker‐Au surface interactions (Figure 3 a), the latter stabilizing the observed final “lying down” conformation for the *PyrMT* ligand.

### Single‐Molecule charge transport of the *(Co‐)P/PyrMT* and *(Co‐)P/PyrT* assemblies

**Figure 4:**
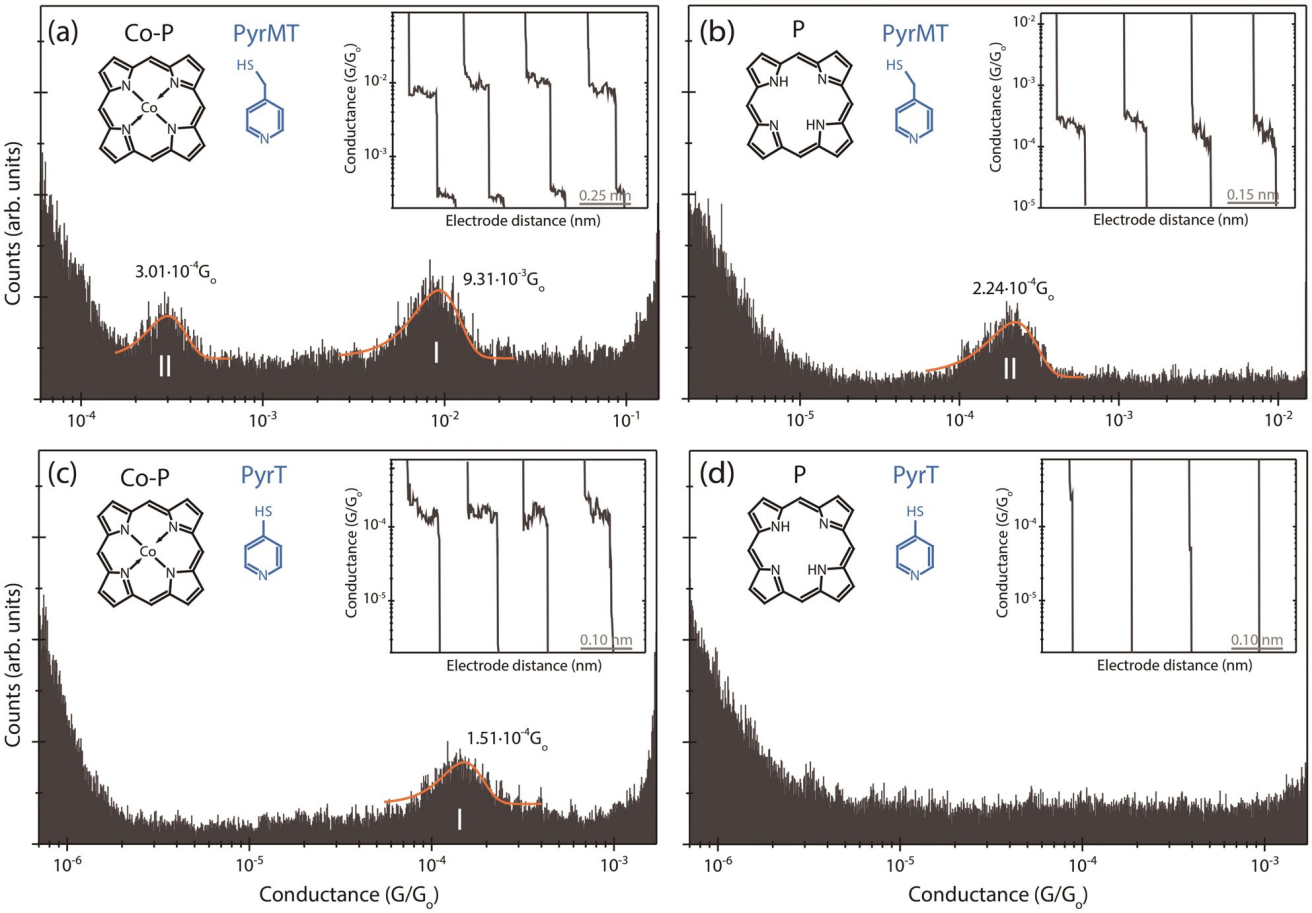
1D semi‐log conductance histograms of the *Co‐P/PyrMT* (a), *P/PyrMT* (b), *Co‐P/PyrT* (c), and *P/PyrT* (d) systems. The most probable conductance values are extracted from Gaussian fits of the peaks. The insets show representative individual current traces used to build the 1D histograms. Counts have been normalized versus the total amount of counts. The applied bias voltages were set to +7.5 mV (a,b,d) and +15 mV (c).

Now we turn our attention to the conductance features II and III in Figures 2 a and b, originating from interactions between the linkers and the porphyrin ring, not involving the metal center. To this aim, we first perform additional experiments using an unsubstituted porphyrin (*Co‐P*) and its metal‐free (*P*) homologous (Figure 4). The *Co‐P* junction using *PyrMT* linkers (Figure 4 a) show two distinguishable molecular conductance signatures I and II at 9.31×10^−3^ G_0_ and 3.01×10^−4^ G_0_ respectively. Same experiment with *P* (Figure 4 b) yields a unique low conductance feature II centered at 2.24×10^−4^ G_0_, close to the equivalent feature II in Figure 4 a, which tentatively leads us to same previous peak assignment: π‐stacked pyridine/metal for peak I (equivalent to signature I in Figure 2) and pyridine/porphyrin ring for peak II interactions (equivalent to either II or III in Figure 2). The overall conductance decreases for all homologous signatures in the *Co‐P* junction as compare to the *Co‐DPP* (see summarizing Table 1), being especially acute for conductance feature II (≈30×). This conductance changes are due to the different phenyl substitution in both *Co‐DPP* and *Co‐P* cases, and they are tentatively ascribed to two different electrical contributions: (i) the phenyl substitution might bring the energy of the HOMO frontier orbital^30^ closer to the Fermi level (see the projected density of states (PDOS) in SI section 5) via electron donation by resonance,^31^ and reduce the energy barrier for the transmitted electrons,^32^, ^33^, ^34^ assuming off‐resonance tunneling through the HOMO level (see also phenyl contribution to the HOMO in Figure S5.8). Such resonance effect is expected to be more pronounced for the pyridine/porphyrin ring interaction (feature II), as experimentally observed. And (ii), the close electrode/phenyl proximity in the highly constrained *Co‐DPP* junctions (Figure 3 d) could also increase the electrode/porphyrin contact area (coupling) giving rise to an increased conductance.

**Table 1:** Summary of most probable conductance values (expressed as 10^−4^ G_o_) of all observed signatures for each porphyrin/linker combinations.

	Porphyrins	*Co‐DPP*			*DPP*			*Co‐P*			*P*		
Linkers	*Peaks*	I	II	III	I	II	III	I	II	III	I	II	III
*PyrMT*		282	97.5	30.2	–	80.8	23.1	93.1	3.01	–	–	2.24	–
*PyrT*		3.5	–	0.17	–	–	0.16	1.51	–	–	–	–	–

The absence of one of the low conductance signatures (II, III) in Figure 4 a when compared to the *Co‐DPP* results with the same linker (Figure 2 a) evidences also the active role of the phenyl porphyrin substitutions in the formation of the supramolecular junction. This pyridine/phenyl interaction is also confirmed by the same measurements performed on junctions based on a 5,15‐dibisphenylporphyrin (*DBPP*), with bi‐phenyl substitutions in the porphyrin ring (see SI section 4.3). The *DBPP* junctions with *PyrMT* linkers display additional conductance signatures (up to 4 overlapping peaks are visible) as compare to the two observed in *DPP* (peaks II and III in SI section 4.1), which demonstrates the additional accessible interaction sites brought by each phenyl ring.

The *Co‐P* junctions employing a *PyrT* linker (Figure 4 c) shows a unique conductance feature whose value is within the same order of magnitude as the conductance signature I in the *Co‐DPP* junctions with the same linker (Figure 2 b), and therefore, ascribed to an axial “lifted” coordination of the pyridine linker to the metal center. The small discrepancy (≈2.3×, see Table 1) in both *Co‐P* and *Co‐DPP* cases is again tentatively ascribed to the electronic donating resonant effect of the phenyl substituents in the porphyrin ring. The absence of conductance signatures II and/or III using *PyrT* linkers for *Co‐P* (Figures 4 c,d) demonstrate that the enhanced flexibility of the *PyrMT* linker, which presents a conductance signature for the junction with the unsubstituted free‐metal porphyrin *P* (Figure 4 b), readily interact with the porphyrin ring thanks to its more accessible π‐π stacking orientation (Figure 3 a). When both the metal center and the phenyl substitutions are removed, the *PyrT* is unable to establish any stable interaction with the porphyrin backbone *P* resulting in a silent conductance histogram (Figure 4 d).

### Computational analysis of the low conductance features II and III

**Figure 5:**
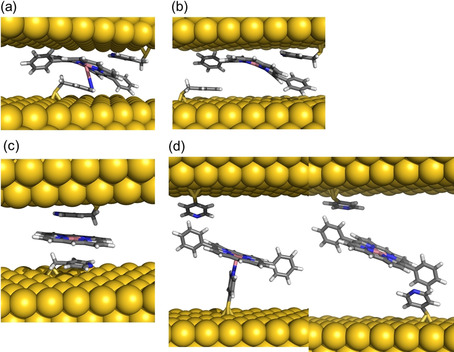
DFT‐optimized structures of the low conductance signatures for the *Co‐DPP*/*PyrMT* system a) labelled as II in Figure 2 a and b) labelled III in Figure 2 a c) the lowest conductance signature for the *Co‐P/PyrMT* system labelled as II in Figure 4 a, and d) two equally probable configurations for the lowest conductance signature of the *Co‐DPP/PyrT* labelled as II in Figure 2 b.

We again computed DFT‐optimized junction geometries to visualize the most plausible interactions that lead to the observed II and III conductance features for the *PyrMT* and *PyrT* linkers, where the metal is partially or not participating in the junction formation. Figure 5 summarizes the minima‐energy DFT junction configurations whose computed conductance values lie within the range of the experimental ones. Starting with the *PyrMT*, Figures 5 a and b show two stable configurations corresponding to the replacement of one and two *PyrMT*‐porphyrin ring interactions, respectively, by *PyrMT*‐phenyl interactions with the *Co‐DPP*. These interactions arise from effective π‐π stacking between the pyridine moiety of the *PyrMT* linker and the phenyl substitutions of the porphyrin. The calculated conductance values for these two optimized geometries are 6.81×10^−3^ and 4.50×10^−3^ G_o_ in agreement with the experimental values of 9.75×10^−3^ and 3.02×10^−3^ G_o_ (Table 1), and they are also consistent with a dynamic picture of consecutive formation of more extended conformations as the junction is elongated during the tip retraction (SI Figure S3.1), where Figure 5 a,b conformations are able to span slightly larger electrode‐electrode gap separations (see SI section 3 for a comparison of the experimental gap separations and the DFT geometries distances of the assemblies). In the absence of phenyl substitutions (*Co‐P* and *P*), we found effective supramolecular interactions between the pyridine moiety of the two *PyrMT* and the pyrrolic ring of the porphyrin (Figure 5 c) characterized by the conductance feature II in Figure 4 a,b. Note that this conformation does not imply interactions with the metal center and that they also seem to be hindered in the *Co‐DPP* case by the presence of the phenyl substitutions, where the pyridine‐phenyl interaction dominates. The computed conductance in Figure 5 c configuration yields 5.5×10^−4^ G_o_, in good agreement with the experimental value for the *Co‐P*/*PyrMT* signature II (Table 1). As for the *PyrT*, the optimized geometries for the *Co‐DPP/PyrT* system suggest two plausible scenarios where either one or two metal/pyridine coordination(s) is(are) replaced by π‐π phenyl‐pyridine interactions which are enabled thanks to the dihedral rotation of the phenyl substituent (Figure 5 d). The calculated conductance values are 4.21×10^−5^ and 1.37×10^−5^ G_o_, respectively, both close to the corresponding experimental III signature,1.71×10^−5^ G_o_ (Table 1). The total DFT energies for both configurations indicate the interaction with two phenyl substituents (Figure 5 d right panel) is 12.6 kcal mol^−1^ more stable suggesting this one as the most plausible scenario for conductance feature III in Figure 2 b. This assumption is also supported by the results corresponding to the free‐metal *DPP*, where the homologous configuration with two phenyl/pyridine interactions (Figure S5.7) is also 17.5 kcal mol^−1^ more stable, leading to a calculated conductance of 1.48×10^−5^ G_o_, close to the experimental value of 1.56×10^−5^ G_o_ (Figure S4.1b). The absence of the peak II signature and of any conductance signature in Figure 4 c and d, respectively is then explained by the inability of the *PyrT* linker to stablish effective π‐π interactions with the pyrrolic ring.

**Figure 6:**
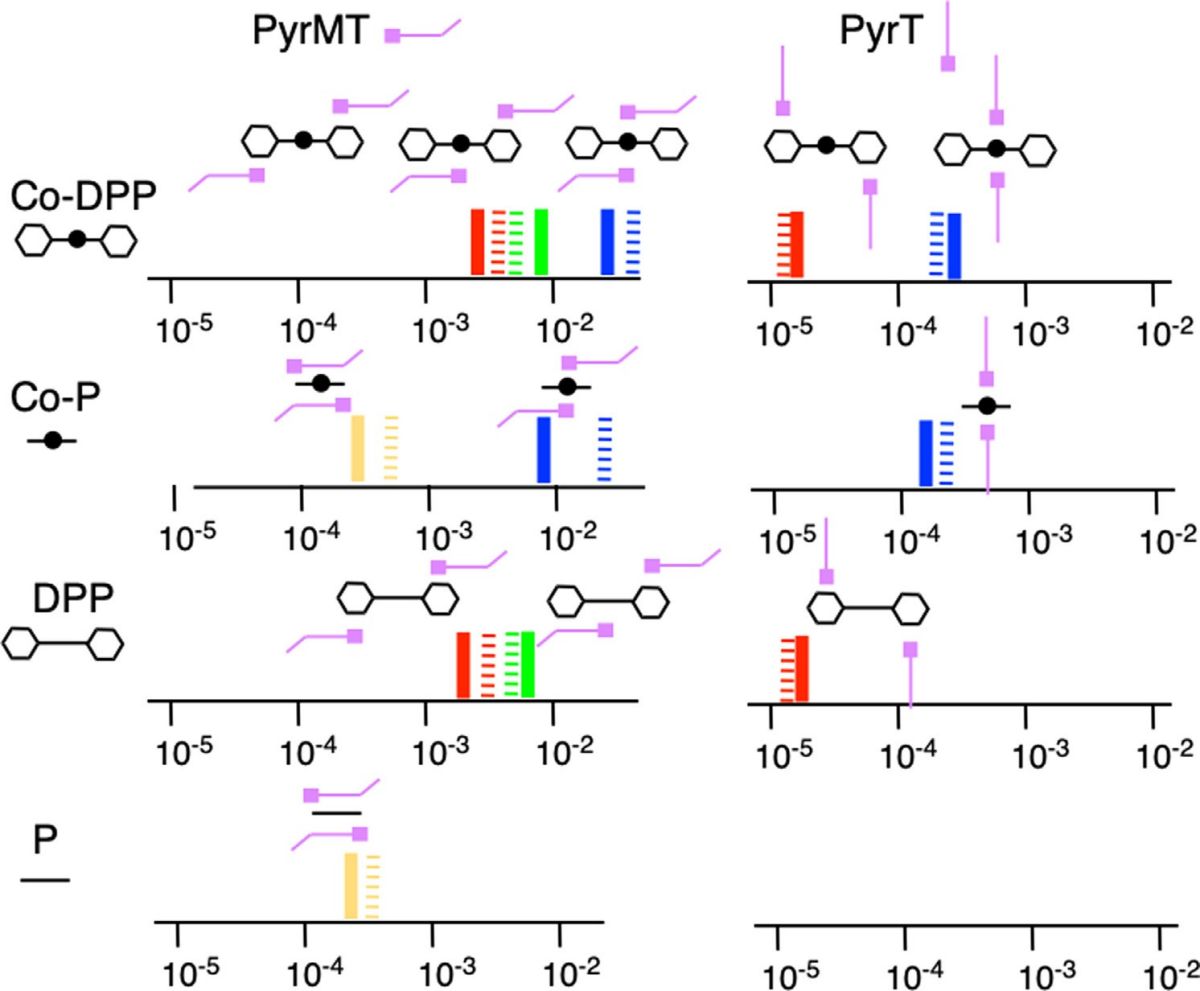
Schematic diagram of the supramolecular landscape for all studied molecular junctions. The conductance values are represented on the *x*‐axis in G_0_ units (solid lines are the experimental values, stripped lines the theoretical values) for both linkers (*PyrMT* and *PyrT*) and the four studied porphyrin systems (*Co‐DPP*, *Co‐P*, *DPP*, and *P*). Schematic representations of the structural models confirmed by DFT are drawn for each conductance signature. The color legend represents equivalent interactions across all junctions: blue corresponds to the both pyridine linkers interacting with the metal center, green is one pyridine interacting with the metal center and the other with a side phenyl group, red is both pyridine interacting with side phenyl rings, and yellow corresponds to two pyridine linkers interacting with the porphyrin pyrrolic ring.

The summarizing Figure 6 maps out all the supramolecular landscape leading to the proposed configurations in our metalloporphyrin‐based single molecule junctions, pinpointing each of the computed supramolecular geometries to every observed single molecule conductance feature. The generally found good agreement between computed and experimental conductance values reinforces the adequacy of DFT+U corrected methods in the studies of supramolecular electronics. Figure 6 picture conceptually opens to new ways of designing nanoscale molecule wires exploiting well‐known supramolecular interactions, paving the way to Supramolecular Electronics. We also expect this work to serve as a platform to study charge transport in biological moieties system exploiting very similar supramolecular interactions to produce scenarios for long‐range electron transfer.

## Conclusion

Concluding, we have studied the formation of single‐molecule electrical contacts in a tunneling junction exploiting the rich axial coordination landscape in metalloporphyrin systems using pyridine‐based linkers. We demonstrate that changes in the linker flexibility result in strikingly different supramolecular interactions between the pyridinic linker and the pyrrolic porphyrin ring, leading to radically different molecular junction geometries. As summarized in Figure 6, an extra methyl group in a *PyrMT* linker, as compare to a *PyrT* linker, confers extra conformational degrees of freedom to the pyridine group resulting in the formation of pyridine/porphyrin π‐π stacking conformations, as opposed to the more orthogonal axial coordination geometries occurring with the more rigid *PyrT* linker. The supramolecular wires conductance resulting from these two distinct geometries differ by two orders of magnitude, being the π‐π stacking conformations more conductive. As the molecular junction is mechanically stretched, more extended supramolecular configurations are identified where either the pyrrolic ring or/and the phenyl side groups of the metalloporphyrin readily provide with additional interacting points to the pyridine linkers, allowing switching to new pathways where the metal center is partially or not participating in the final supramolecular junction structure.

These results demonstrate the large conductance tunability of a molecular wire via tweaking its internal supramolecular interactions and present a novel platform to investigate the fascinating, yet unknown, field of the mechanobiology of electron transport in complex biomolecular structures. This porphyrin‐based supramolecular wire also brings a new synthetic testbench to study the molecular mechanisms supporting the exceedingly long‐range (from mesoscopic to micrometer range) charge transport in bacteria nanowires exploiting similar supramolecular interactions.^35^

## Conflict of interest

The authors declare no conflict of interest.

## Supporting information

As a service to our authors and readers, this journal provides supporting information supplied by the authors. Such materials are peer reviewed and may be re‐organized for online delivery, but are not copy‐edited or typeset. Technical support issues arising from supporting information (other than missing files) should be addressed to the authors.

Supplementary
