# Supplementary material for: Tuning Single‐Molecule Conductance in Metalloporphyrin‐Based Wires via Supramolecular Interactions
Source: Angew Chem Int Ed Engl. 2020 Aug 24;59(43):19193–201. doi: 10.1002/anie.202007237 (PMC7590179; doi:10.1002/anie.202007237)
Supplement: Supplementary file 1 — Supplementary [file ANIE-59-19193-s001.pdf]

Angewandte Chemie
Eine Zeitschrift der Gesellschaft Deutscher Chemiker

Supporting Information

Tuning Single-Molecule Conductance in Metalloporphyrin-Based Wires via Supramolecular Interactions

Albert C. Aragonès, Alejandro Martín-Rodríguez, Daniel Aravena, Josep Puigmartí-Luis, David B. Amabilino, Núria Aliaga-Alcalde, Arántzazu González-Campo, Eliseo Ruiz, and Ismael Díez-Pérez**

anie_202007237_sm_miscellaneous_information.pdf

Supporting Information

Table of Contents

1. **Characterization of linkers-functionalized electrodes (XPS and ellipsometry)**............ 1
2. **2D conductance histograms**.. 3
3. **Pulling curves and plateau length histograms**.. 4
4. **Control single-molecule measurements**.. 6

4.1. Free-metal porphyrins (DPP) measurements 6

4.2. Pyridinyl-functionalized and unfunctionalized electrodes measurements 7

4.3. 5,15-dibisphenylporphyrin measurements (DBPP).. 7

5. **Computational results**... 8
6. **Technical details of the single-molecule transport measurements**............................... 14
7. **Synthesis of compounds**.. 15
8. **References** 19

1. Characterization of linkers-functionalized electrodes (XPS and ellipsometry)

Pyridine-4-yl-methanethiol (*PyrMT*) and 4-Pyridinethiol (*PyrT*) electrodes functionalization was performed using ethanolic solutions as previously reported (see SI Section 6 for sample preparation).[1,2] Previous surface-functionalized *PyrT* studies report the decomposition of *PyrT*-based monolayers on Au in ethanol related to the presence of O_2.[3] To keep the functionalized Au surface in anaerobic conditions, the Au substrate is annealed and preserved under inert N_2 atmosphere, and the compound-containing solutions is N_2 purged and preserved under N_2 atmosphere before and during the functionalization process. To avoid the photo-generation of radical species,[3,4] the functionalization as well as the STM measurements were carried out minimizing direct light exposure. The employed *PyrT* and *PyrMT* were thoroughly purified to avoid the presence of atomic S since its adsorption on the Au surface competes with linkers adsorption.[1]

The X-ray photoelectron spectroscopy (XPS) and ellipsometry data indicate that our self-assembly procedure for *PyrT* molecules produced a fairly stable self-assembled monolayer when immersed overnight, and confirming the stability of the S−Au(111) for both *PyrT* and *PyrMT* functionalization. The S, C and N high-resolution XPS show S:N proportion 3:1 (Fig. S1.1), demonstrating the remanence of significant amounts of 4-pyridinethiol on the Au surface.

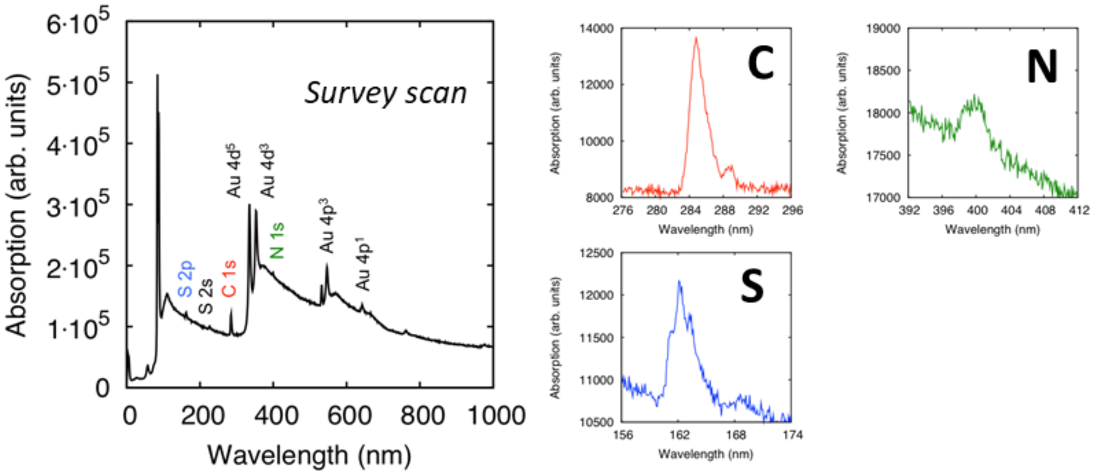

Figure S1.1. X-Ray photoelectron spectroscopy survey scan of the *PyrT*-functionalized Au(111) surface (left) and the high-resolution scans for C, N and S elements (right). The latter shows that a significant amount of monolayer did not decompose.

The ellipsometry measurements were done with an alpha-SE Ellipsometer from J.A. Woollam Ellipsometry Solutions. We acquired data with a wavelength range of 380-900 nm and angles of incidence of 65, 70 and 75º. The monolayers are modeled as a Cauchy optical layer with an Urbach absorption tail on a Au(111) and fitted with the CompleteEASE software to obtain the layer thickness. We have used ellipsometry to characterize whether the *PyrT* and *PyrMT* linkers are in a "tilted" or "lying-down" geometry, as calculations suggest, when interacting with the *Co-DPP* and Au(111) surface. To check this, we have prepared a solution of 3 mg of *Co-DPP* and 2 mg of the ligand in 20 mL of $HCCl_3$. The solubility of *Co-DPP* is rather low at room temperature, thus we filter the solution. The solution is about $1{\cdot}10^{-4}$ M of the octahedral complex, being the employed ligand in excess. Ellipsometry measurements have been done after dipping a Au(111) single crystal during 20 min in a *Co-DPP*/linker solution and dried afterwards under dry N_2. (Figure S1.2 for both ligands).

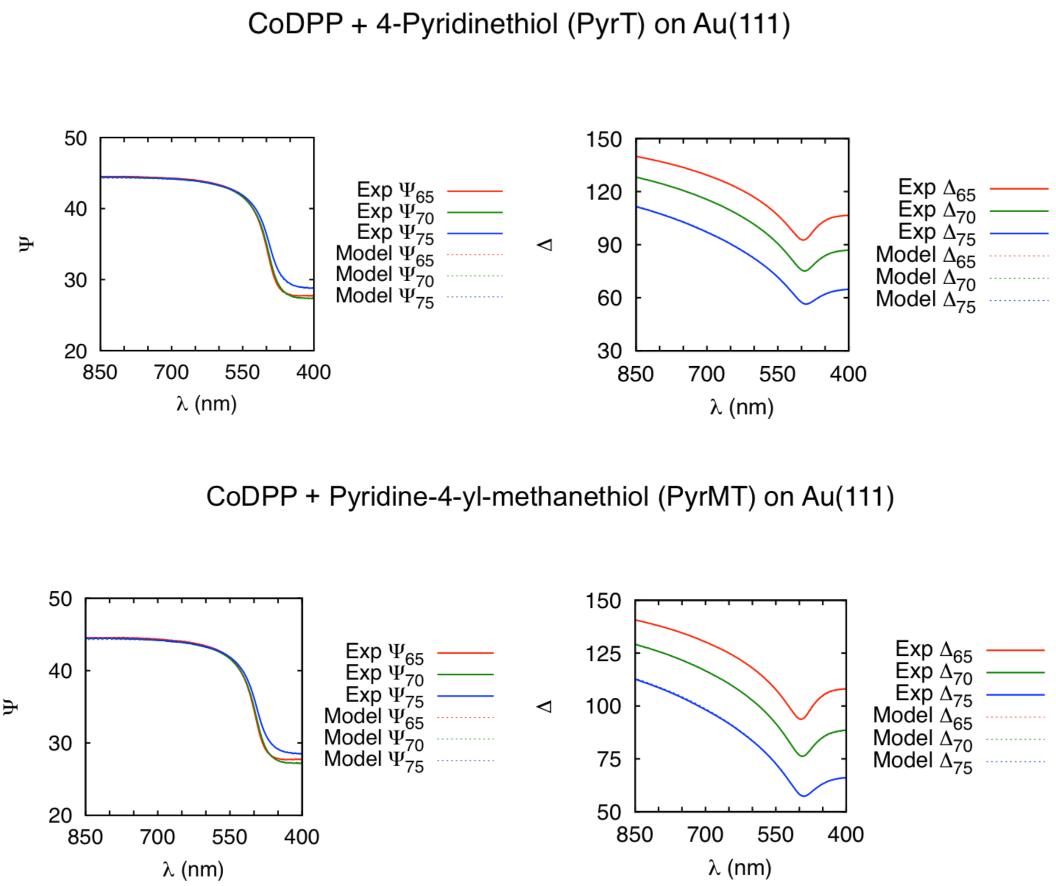

Figure S1.2. Top panel: ellipsometry data for the *Co-DPP/PyrT* monolayer. The monolayer is modelled as a Cauchy layer with equation: n(λ)=1.754 ± 0.025 and an Urbach absorption tail of k=0.202 ± 0.008 with MSE = 2.451. Bottom panel: ellipsometry results for *Co-DPP/PyrMT* monolayer. The monolayer is modelled as a Cauchy layer with equation: n(λ)=1.516 ± 0.042 and an Urbach absorption tail of k=0.156 ± 0.012 with MSE = 2.889.

We obtain a 13.0 ± 0.3 Å layer thickness for the *Co-DPP/PyrT* monolayer and a 11.6 ± 0.6 Å thickness for the *Co-DPP*/*PyrMT* using same fitting scheme. The thicknesses are in good agreement with a Au/*PyrMT*(lying down)/*Co-DPP* and a Au/*PyrT*(lifted)/*Co-DPP* monolayers, supporting our hypothesis (Fig. 3c and main text discussion).

2. 2D conductance histograms

2D histogram S2.1a shows the uneven correlation in the sequence of appearance of the junction geometries represented by conductance features I to III for the *Co-DPP/PyrMT* system. On average, feature I correlates well with both features II and III, while correlation of the latter two is much lower, showing up in the 2D histogram as non-consecutive events. Out of the total 3066 curves accumulated in the 2D histogram S2.1a, 563 (18.4%) displayed clean conductance plateau features I-III (see overlaid individual traces in S2.1), which were selected to build the 1D histogram shown in Fig. 2a. Of these traces, 296 (65%) show consecutive features I and III (Fig. S3.1a) and 150 (33%) show

consecutive features I and II (Fig. 3.1b). Only 2% displayed the three I-III features in a single trace (inset Fig. 2a). In conclusion, the dynamic picture arising from the S2.1a histogram shows that more stable supramolecular adduct I (Fig. 3d) forms first at very short gap separations, as witnessed by its significantly longer plateau length (see SI section 3). The structure then evolves to either the more extended supramolecular adduct II or III (of similar lengths, see SI section 3) as the gap distance expands. Conductance features I and III in the *Co-DPP/PyrT* system appear mostly consecutively correlated (Fig. S2.1b).

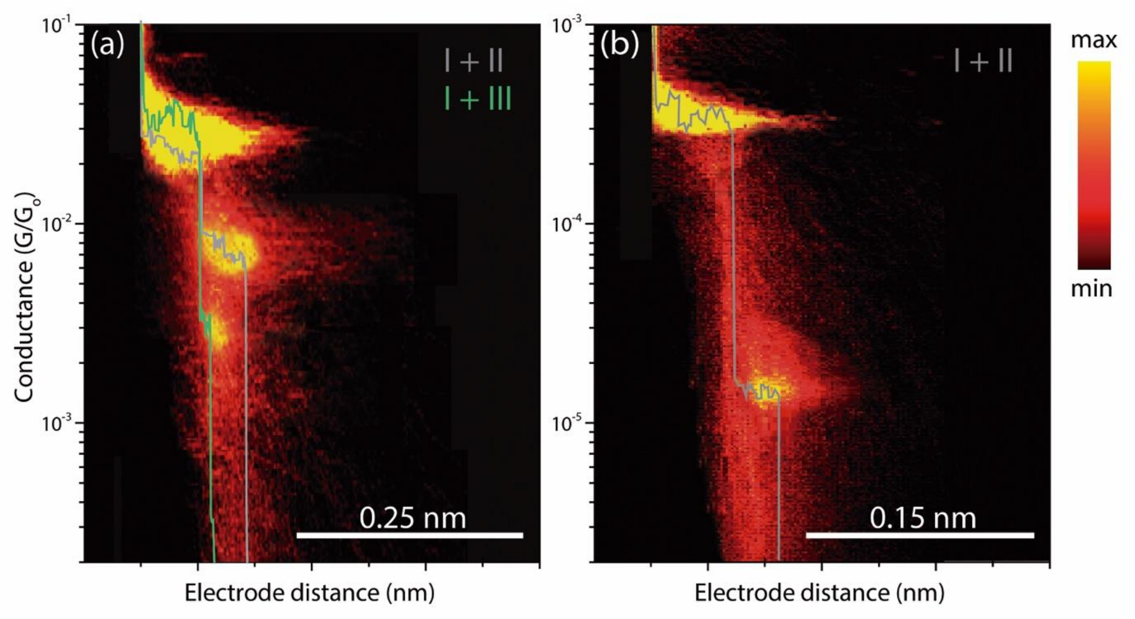

Figure S2.1. 2D conductance semi-log maps built out of several thousands of individual current traces with no data selection for the *Co-DPP* junctions using *PyrMT* (a) and *PyrT* (b). The applied bias voltage was set to 7.5 mV.

3. Pulling curves and plateau length histograms

Figure S3.1 pinpoints the three observed conductance signatures I to III to their assigned supramolecular geometries for the *Co-DPP/PyrMT* system. Consecutive plateaus in the individual current traces pairing in a I-II and I-III fashion evidence the switching between the proposed supramolecular structure I to either structures II or III as correlated in previous section 2. The calculated energies for the I to III interactions show an excellent agreement with the plateau length of the corresponding conductance feature (see Fig. S3.2 for all the tested systems). The final electrode-electrode separations of the DFT relaxed structures, 9.36, 10.5 and 10.7 Å for features I, II and III respectively, follow well the experimental trend 6.98, 8.0 and 8.14 Å, where a ~0.5 nm gold snap-back has been added[5]. The small discrepancy in the gap separation values might manifest the out-of-equilibrium nature of the dynamic break-junction in the experiments as opposed to the complete relaxed structures arising from the DFT optimization. The good correlation in the trend though suggests agreement on the calculated geometries to the structures being formed during the dynamic supramolecular wire formation.

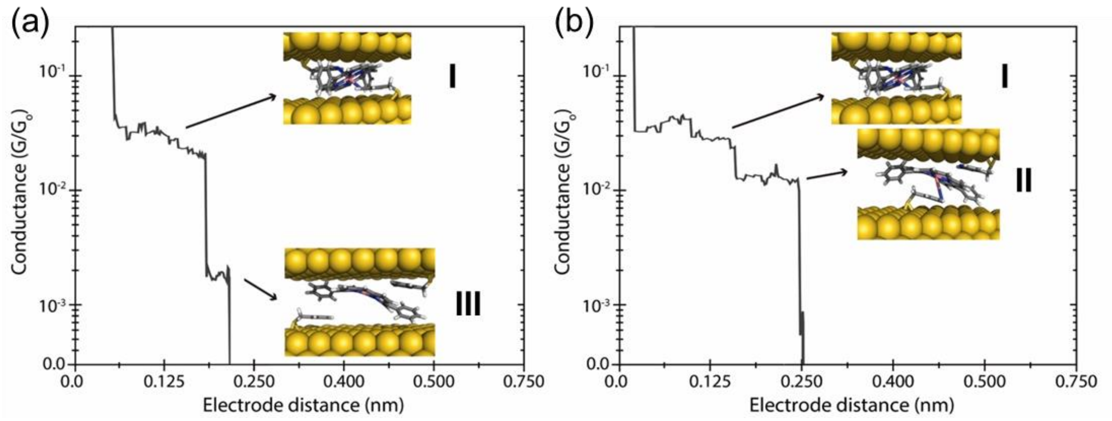

Figure S3.1. Two representative individual traces for the *Co-DPP/PyrMT* system showing the two most likely correlations with (a) the I and II features and with (b) the I and III features. The DFT-optimized structures are depicted in the figure inset.

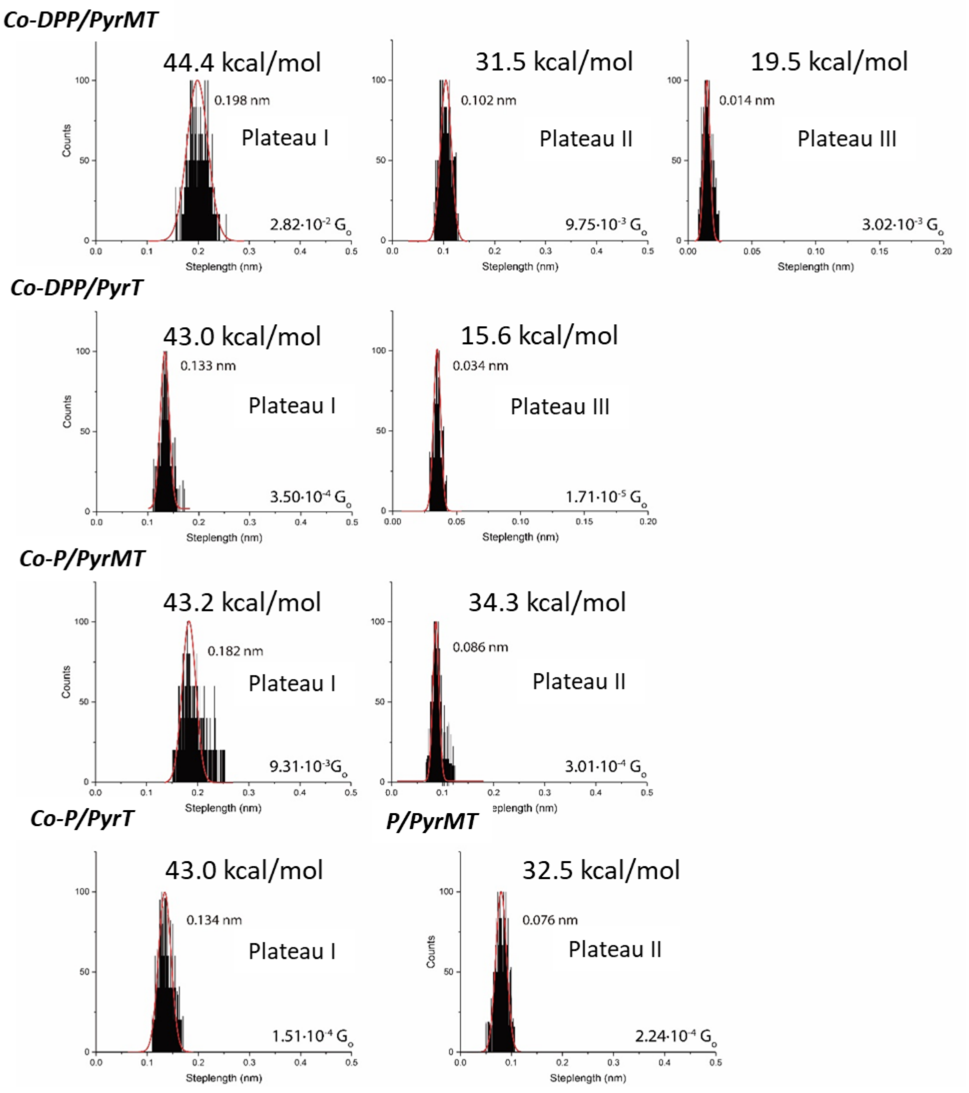

Figure S3.2. Plateau length histograms of the main observed conductance features in Table I. Inset info: plateau length (in nm, from Gaussian fit), DFT structure energy (in

kcal/mol), average conductance plateau (in G_0 scale) and conductance plateaus labeled I-III.

4. Control single-molecule measurements

4.1 *Free-metal porphyrins (DPP) measurements*

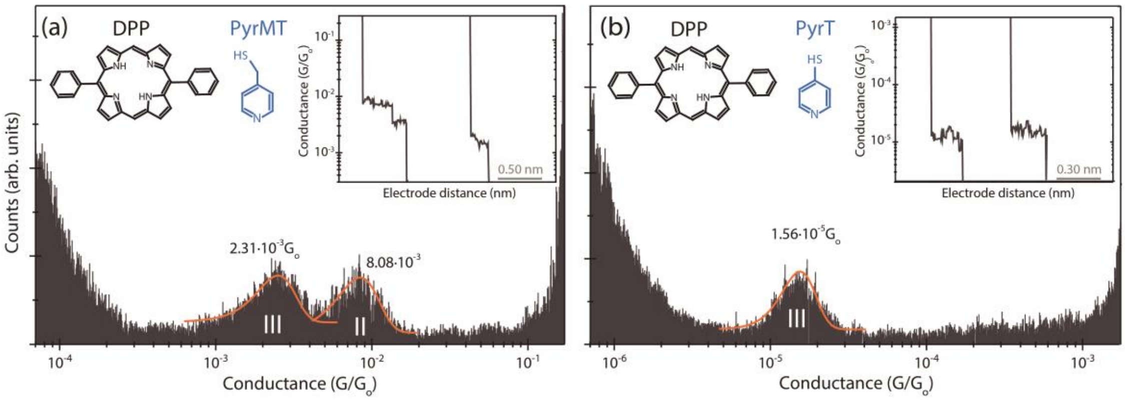

Figure S4.1. 1D semi-log conductance histograms of the *DPP*/*PyrMT* (a) and *DPP*/*PyrT* (b) systems. The conductance values are extracted from Gaussian fits of the peaks. The insets show representative individual current traces displaying plateau features used to build the 1D histograms. Like the *Co-DPP/PyrMT* case (Fig. 2), feature II was found in less than 20% of the traces displaying feature III, showing again poor correlation between both structures. Counts have been normalized versus the total counts number. The applied Bias voltages were set to +7.5 mV.

4.2 *Pyridinyl-functionalized and unfunctionalized electrodes measurements*

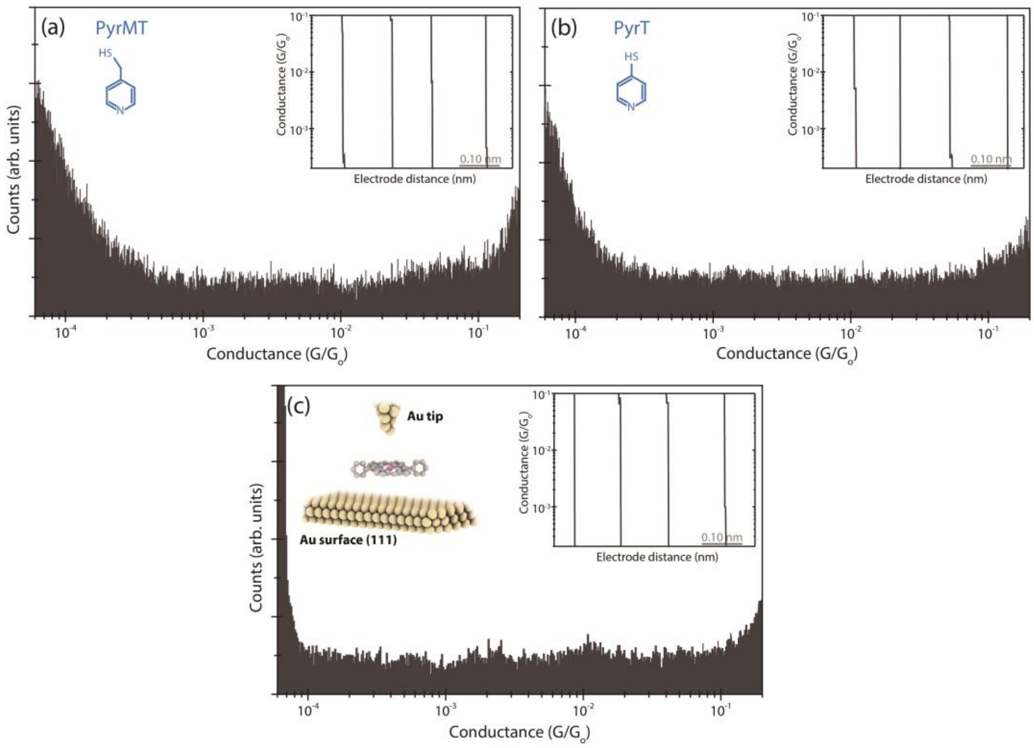

Figure S4.2. 1D semi-log conductance histograms for measurements in absence of porphyrin with both tip and surface functionalized with *PyrMT* (a) and *PyrT* (b). 1D semi-log conductance histograms for measurements without linker at both electrodes when *Co-DPP* is present in solution (c). The insets show representative individual traces used to build the 1D histograms. The low counts background observed in (c) is the result of electrode-porphyrin unspecific interactions. Counts have been normalized versus the total counts number. The applied Bias voltages was set to +7.5 mV.

4.3 *5,15-dibisphenylporphyrin measurements (DBPP)*

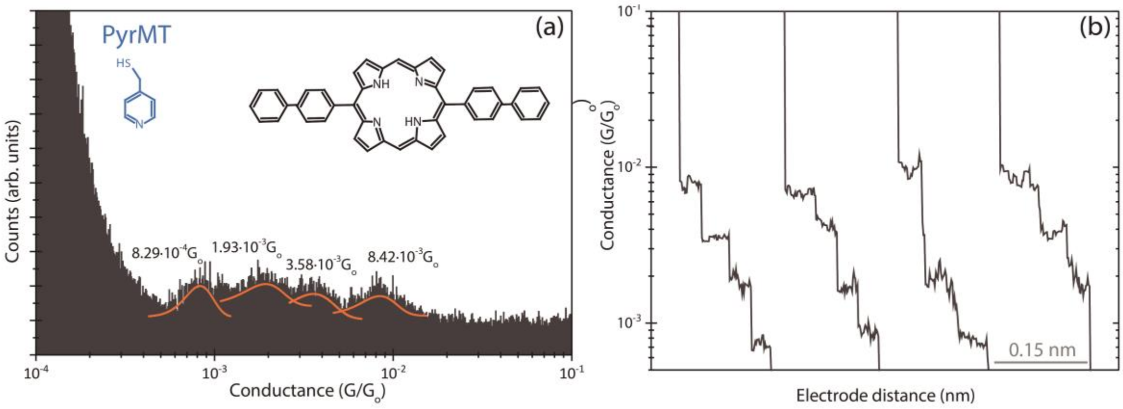

Figure S4.3. (a) 1D semi-log conductance histograms of the *DBPP*/*PyrMT*. The conductance values are extracted from Gaussian fits of the peaks. Counts have been normalized versus the total counts number. (b) Representative individual current traces displaying plateau features used to build the 1D semi-log histograms in (a). The applied Bias voltages was set to +7.5 mV.

5. Computational results

Electron transport calculations were carried out with the molecule sandwiched between five Au layers with a 5 x 4 surface unit cell using the Siesta[6] and Gollum[7] codes with the GGA[8]+U functional (U = 4.0 eV) using the exchange-correlation functional proposed by van Voorhis[9,10] and coworkers to include dispersion effects. The +U approach was employed to have semiquantitative conductance values thanks to the better description of the energy of the frontier orbitals. A double-ζ basis set with polarization was used combined with pseudopotentials. For Au atoms, two pseudopotentials have been employed; 11 e^- pseudopotential for optimizations and 1e^- for the transport calculations[11]. For the Co atom, a semi-core pseudopotential was used, thus the 3p orbitals were considered within the basis sets. To obtain the conductance value, we approximate the conductance $G = T(E_F)G_0$ which should be suitable for the employed low experimental Bias voltages. To compare the PBE results obtained with Siesta and Gollum against a hybrid functional, Artaios code was used to calculate the transport properties within the Wide Band Limit (WBL) approximation. The electronic structure was obtained using Gaussian code with the B3LYP functional and the LANL2DZ basis set.

In the case of the geometry optimization of the two ligands (*PyrMT* and *PyrT*) on the Au surface, the comparison of the relative energies for lying-down and standing-up conformations is a difficult case for pair dispersion models such as the van Voorhis functional[9]. Hence, the calculations were performed with a more accurate many-body approach[12] implemented in the FHI-AIMS code[13] using the PBE functional and the tight basis set[14,15]. The structures of the porphyrins interacting axially with the linkers (main Figs. 3 and 5) were obtained from DFT structure optimizations using the Siesta code.

In all studies, a 3-fold hollow Au-S bond was defined as the most stable contact configuration. Changes in Au-S contact geometries were not observed in our experimental data where the appearance of conductance features I-III depends exclusively on the presence of porphyrin chemical substitutions such as the metal centre and/or the phenyl side groups. Different thiol contact configurations were therefore not considered in the calculations.

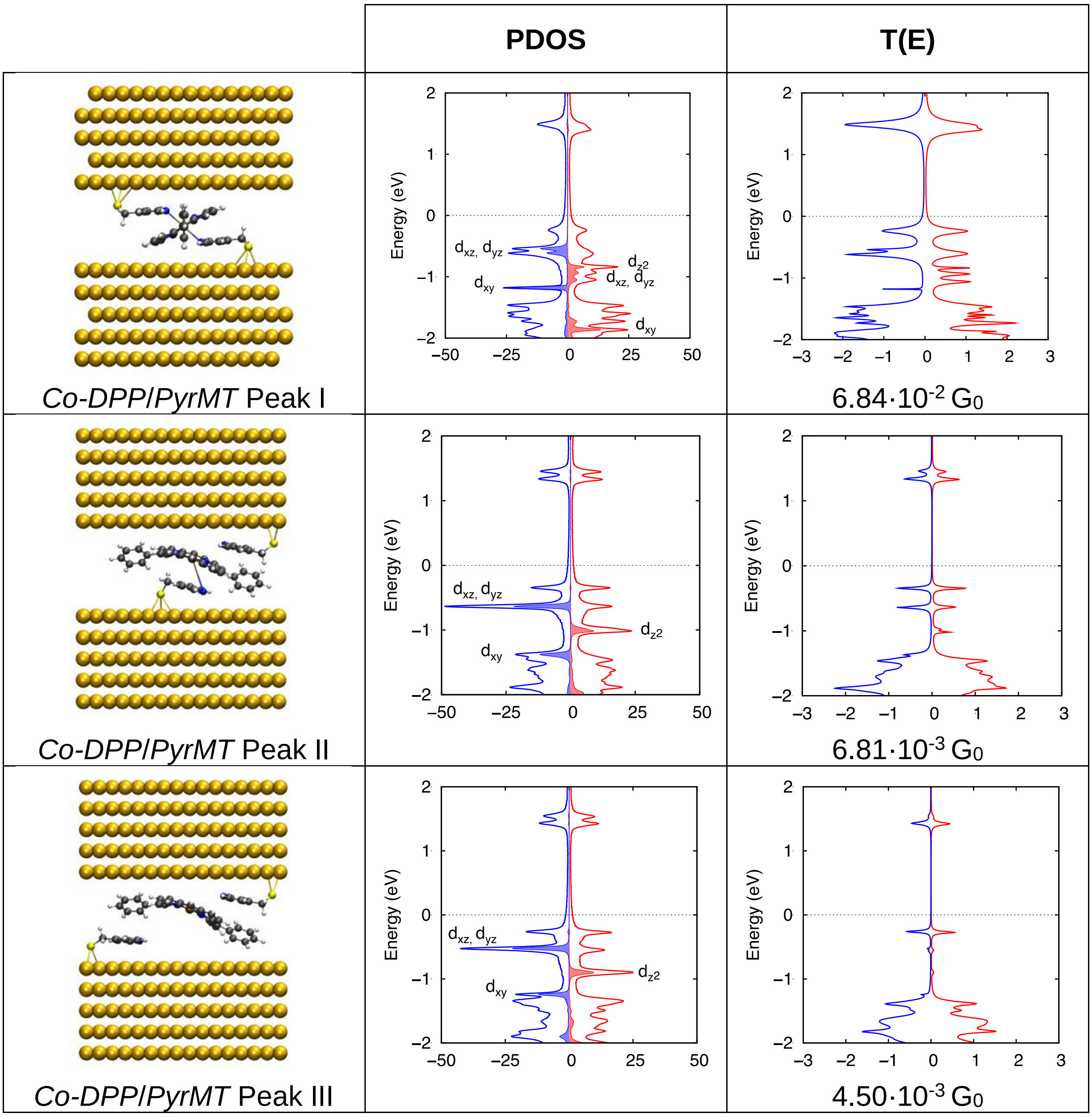

Figure S5.1. Optimized structures (left column), projected density of states (PDOS) with shaded regions corresponding to metal projection (central column), transmission curves and zero-energy calculated G values (right column) for the *Co-DPP/PyrMT* system using the Siesta and Gollum codes with the GGA+U functional (U = 4.0 eV) and the exchange-correlation functional proposed by van Voorhis *et al.*[9] to include dispersion effects.

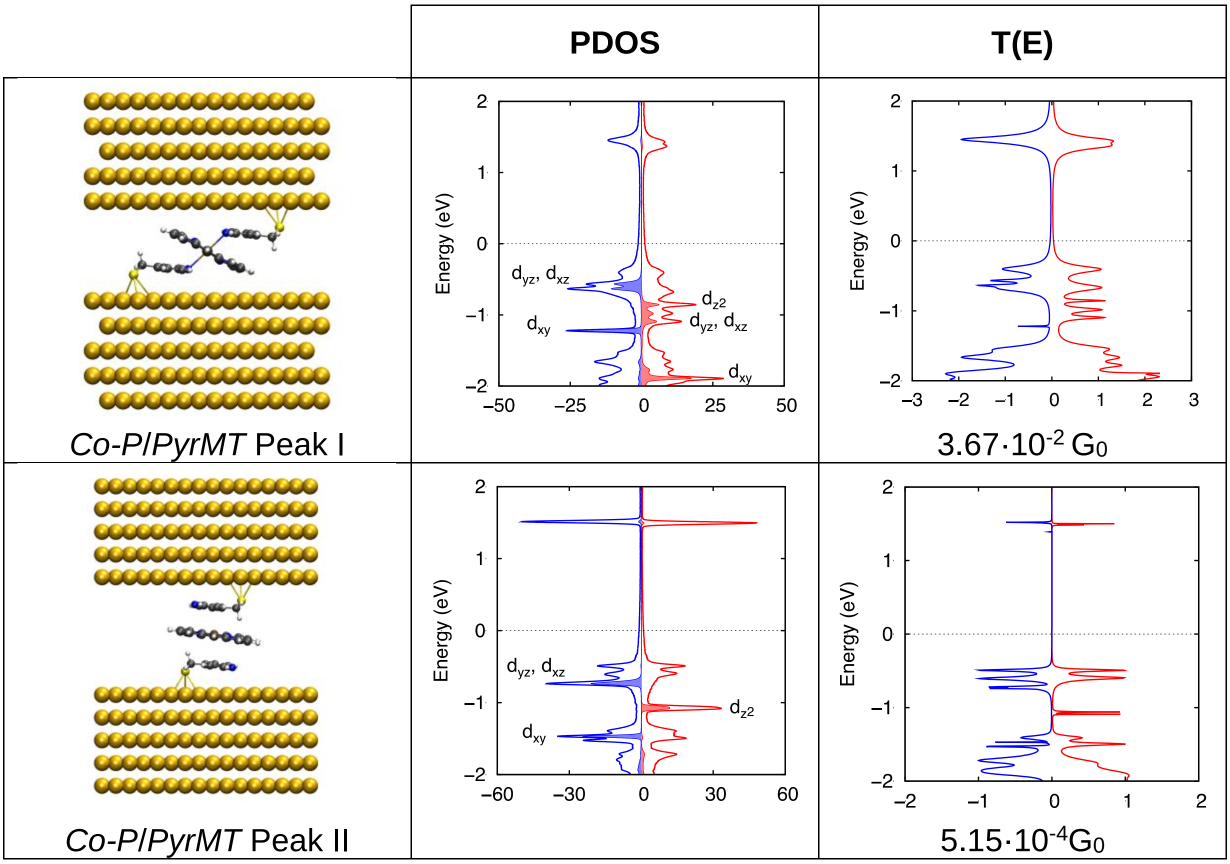

Figure S5.2. Optimized structures (left column), PDOS with shaded regions corresponding to metal projection (central column), transmission curves and zero-energy calculated G values (right column) for the *Co-P/PyrMT* system using the Siesta and Gollum codes with the GGA+U functional (U = 4.0 eV) and the exchange-correlation functional proposed by van Voorhis *et al*.[9] to include dispersion effects.

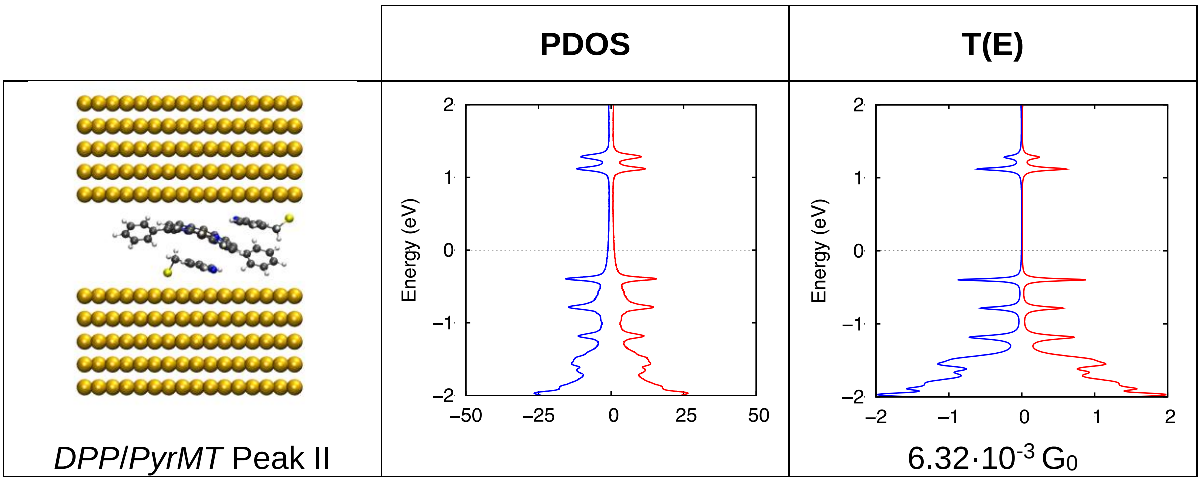

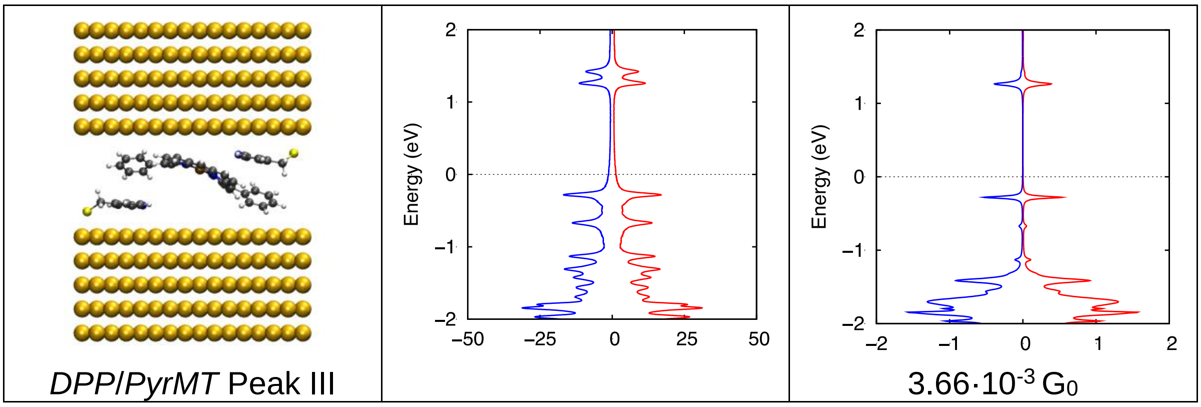

Figure S5.3. Optimized structures (left column), PDOS (central column), transmission curves and zero-energy calculated G values (right column) for the *DPP/PyrMT* system using the Siesta and Gollum codes with the GGA+U functional (U = 4.0 eV) and the exchange-correlation functional proposed by van Voorhis *et al.*[9] to include dispersion effects.

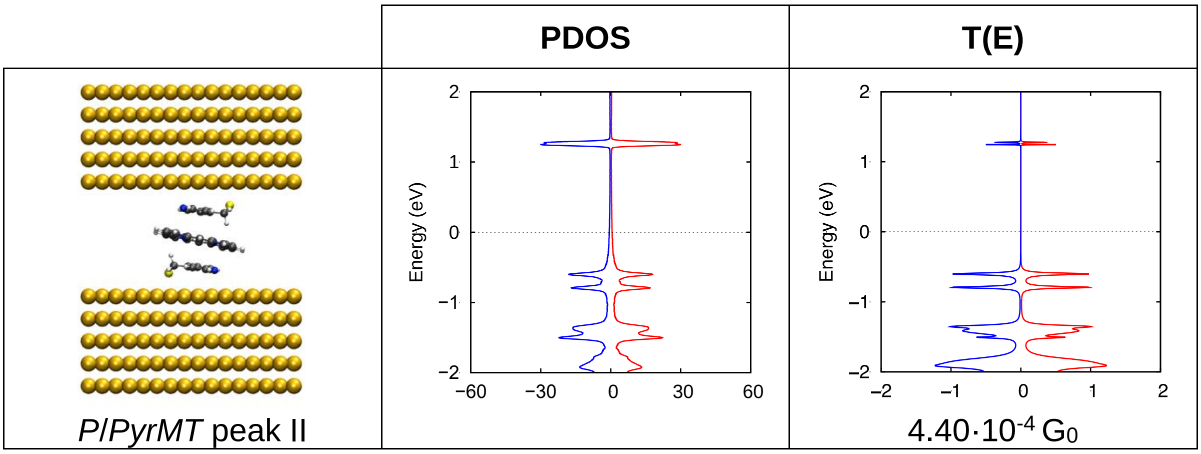

Figure S5.4. Optimized structures (left column), PDOS (central column), transmission curves and zero-energy calculated G values (right column) for the *P/PyrMT* system using the Siesta and Gollum codes with the GGA+U functional (U = 4.0 eV) and the exchange-correlation functional proposed by van Voorhis *et al.*[9] to include dispersion effects.

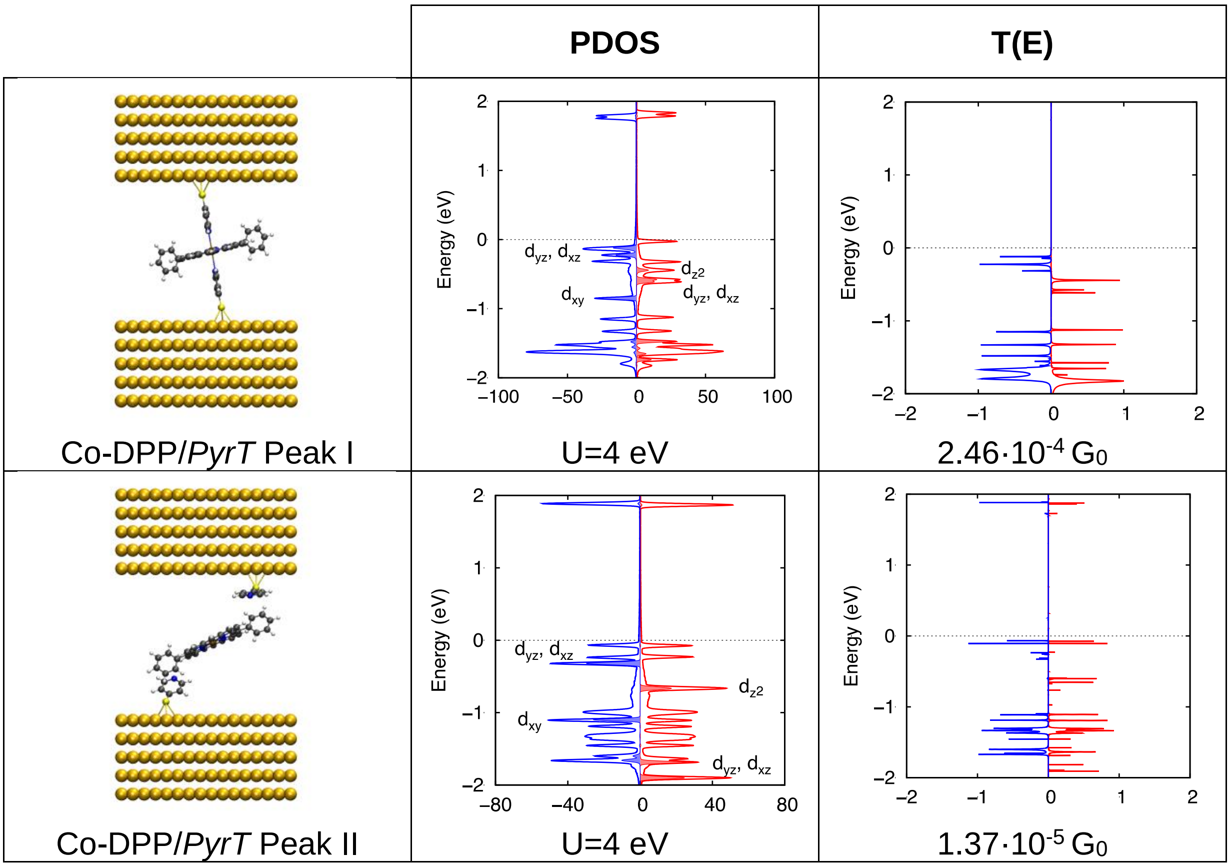

Figure S5.5. Optimized structures (left column), PDOS with shaded regions corresponding to metal projection (central column), transmission curves and with zero-energy calculated G values (right column) for the *Co-DPP/PyrT* system using the Siesta and Gollum codes with the GGA+U functional (U = 4.0 eV) and the exchange-correlation functional proposed by van Voorhis *et al.*[9] to include dispersion effects.

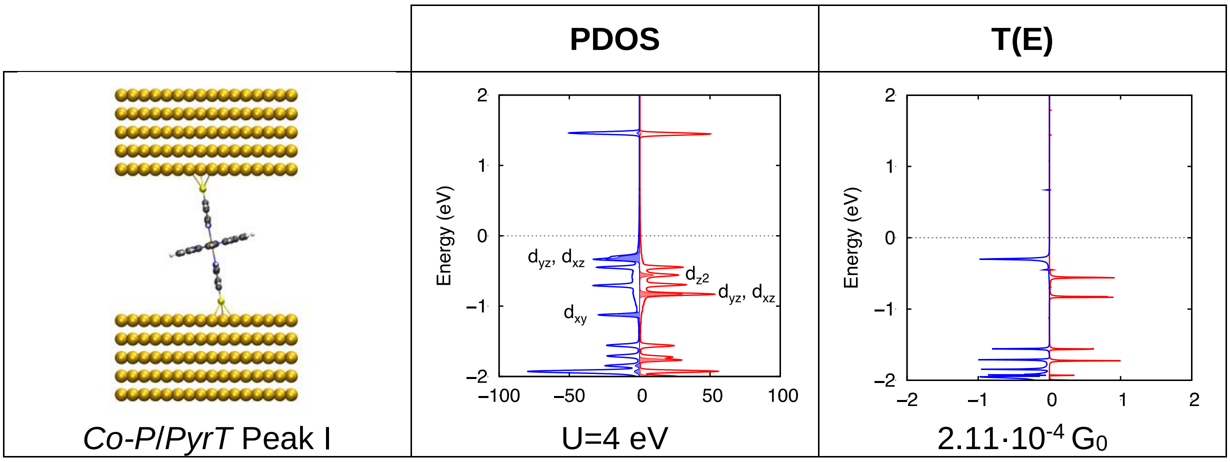

Figure S5.6. Optimized structures (left column), PDOS with shaded regions corresponding to metal projection (central column), transmission curves and with zero-energy calculated G values (right column) for the *Co-P/PyrT* system using the Siesta and

Gollum codes with the GGA+U functional (U = 4.0 eV) and the exchange-correlation functional proposed by van Voorhis *et al.*[9] to include dispersion effects.

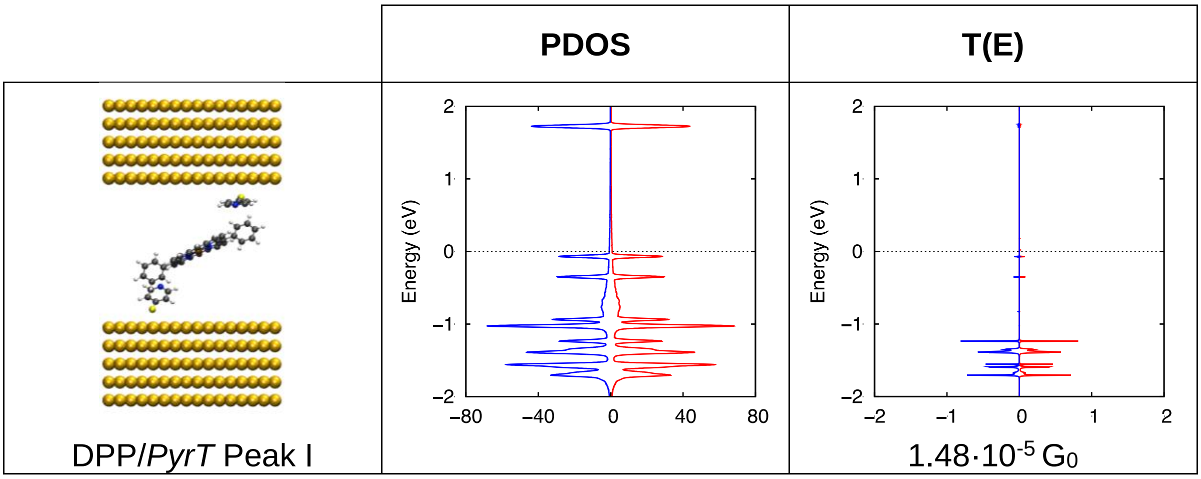

Figure S5.7. Optimized structure (left column), PDOS (central column), transmission curve and zero-energy calculated G values (right column) for the *DPP*/*PyrT* system using the Siesta and Gollum codes with the GGA+U functional (U = 4.0 eV) and the exchange-correlation functional proposed by van Voorhis *et al.*[9] to include dispersion effects.

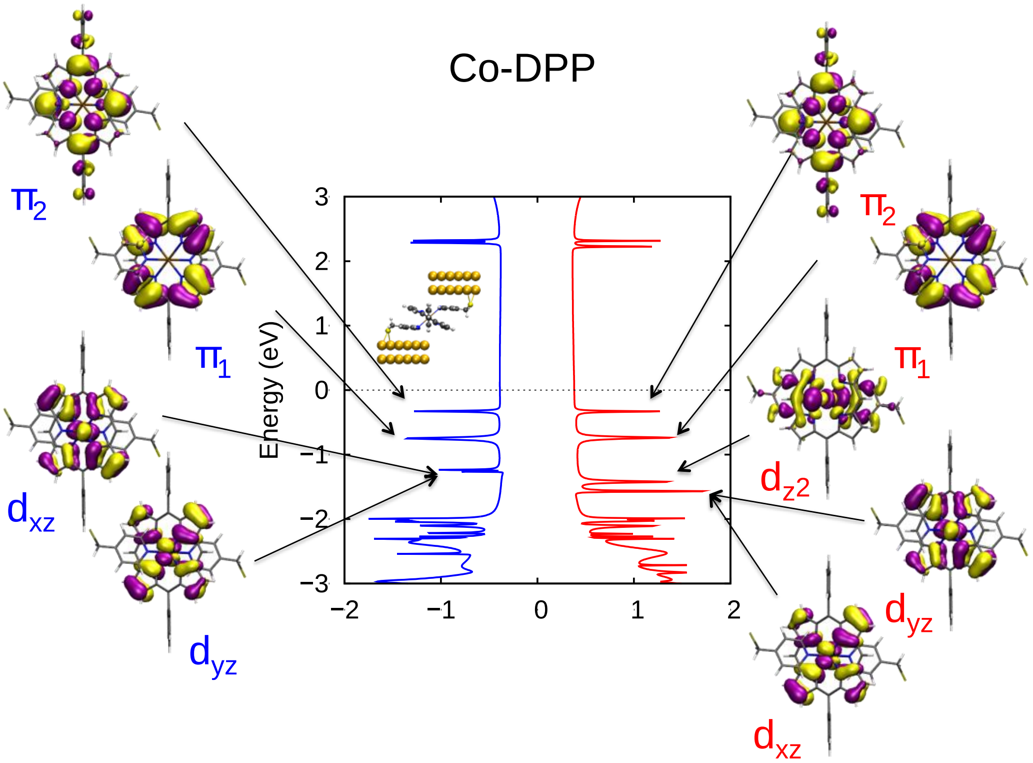

Figure S5.8. Model structures and transmission curves using the Gaussian and ARTAIOS codes with the B3LYP[16] functional and LANL2DZ[17–20] basis set. The geometries are

the ones obtained in the optimization with Siesta, but the gold electrodes are modified in order to reduce the computational cost.

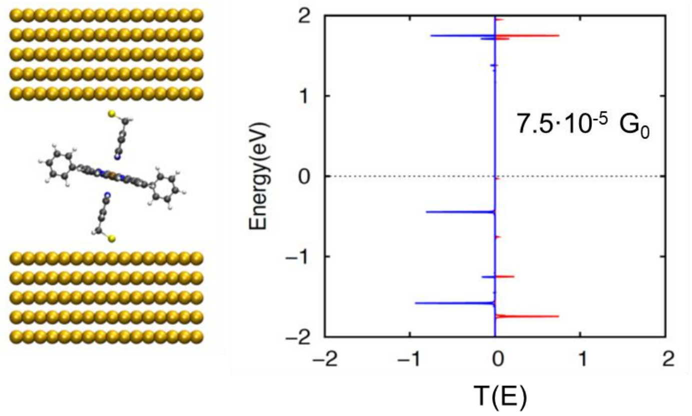

Figure S5.9. Optimized structure and transmission curves for a hypothetical *PyrMT*(standing up)/Co-*DPP*/*PyrMT*(standing up) junction geometry. The integrated zero-energy conductance value as graph inset.

6. Technical details of the single-molecule transport measurements

Single-molecule experiments. The details of the STM-break junction technique have been published elsewhere.[21,22] All the conductance measurements were carried out with a mechanically and electronically isolated PicoSPM II microscope head controlled by a Picoscan-2500 electronics (all from Keysight) and using a homemade PTFE-STM cell. Data captures were acquired using a NI-DAQmx/BNC-2110 National Instruments (LabVIEW data acquisition System) and analyzed with LabVIEW code. In a typical break-junction experiment, the STM tip is brought to tunneling distance over a flat clean Au (111) surface area as a first step. The STM feedback is then turned off and the tip is driven into and out of contact with the substrate at a speed of ~2 nm/s. This 2-points feedback loop is used to capture thousands of current decays (~3000-4000). Single molecule conductance (G) was determined using the expression $G=I_{plateau}/V_{Bias}$, where I is the current and V is the voltage difference between the two junction electrodes. Selected current decays displaying molecular plateau features are accumulated to semi-logarithmic conductance histograms. The observed plateaus in the individual current decays result in the observed peaks in the conductance histograms and provide most probable values of the single-molecule conductance. Simple algorithms built in a LABVIEW code are used to identify traces bearing clean plateaus based on a few straightforward criteria: (1) a maximum total decay time identifying traces from clean Au-Au breakdowns only, (2) minimum counts number in any given data bin along the current decay identifying plateaus, and (3) maximum current spike tolerance to reject noisy traces masking any molecular feature. The histograms were compiled by applying the same automated selection criteria across all experimental series. The percentage decay curves that showed clear molecular steps (fulfilling the above criteria) were typically 15–20% and were all

selected to build the histograms.[23–25] This selection process made peaks in the 1D conductance histograms more prominent above the tunneling background and also allowed a quantitative measure of the yield of molecular junction formation in all conductance measurements. Contrarily, 2D histograms were obtained without any selection criteria.

Samples preparation. All glassware and PTFE-STM cells were cleaned with piranha solution (13:1 H_2SO_4/H_2O_2 by volume) before usage followed by thoroughly rinsing with 18 MΩ cm^{-1} Milli-Q water (Millipore). An Au (111) single crystal substrate (10 mm x 1 mm) of 99.9999% purity and orientation accuracy < 0.1 degrees was purchased from MaTeck (Germany). Before each experiment, the single crystal Au (111) substrate was electropolished to eliminate possible residual contamination and then annealed with a H_2 flame. Both STM probe and substrate surfaces were immediately immersed in an Ar purged 5 mM ethanol solution of pyridin-4-yl-methanethiol (or 4-Mercaptopyridine) for 24 h. The Au (111) surfaces and Au tip were then washed thoroughly with ethanol and dried under a stream of argon. The Au (111) surface was then assembled in the STM cell and the STM cell filled with a 80 µL of pure mesitylene, and STM junction control experiments were run first. Next, few drops of a 10 nM mesitylene solution of the porphyrin were added and measurements repeated to study the porphyrin-based molecular wires.

7. Synthesis of compounds

Pyridin-4-yl-methanethiol was synthesized as described previously by Puigmarti-Luis et. al.[26] (Fig. S7.4). *DPP* was purchased from Frontier Scientific and was used as received (Fig. S7.7), and *Co-DPP* was synthesized according to the published procedure described by Song et. al.[27] and purified by column chromatography and crystallization (Figs. S7.6). The 4-mercaptopyridine was purchased from Sigma-Aldrich and used as received (Fig. S7.5). Dipyrromethane was synthesized as described previously by Linsey *et al.*[28]

Synthesis of 5,15-dibisphenylporphyrin (DBP, Figs. S7.1-7.3)

Dipyrromethane (150 mg, 1.05 mmoL) and biphenyl-4-carboxaldehyde (181 mg, 0.99 mmoL) were dissolved in dry CH_2Cl_2 (100 mL) under Ar. Then, 25 µL of trifluoroacetic acid were added and the mixture was stirred for 4 hours. After this time 0.320 g of p-choranil (1.30 mmol) were added and the mixture was refluxed for 1 hour. Then, the mixture was filtered through celite and the volatiles were removed under vacuum. The solid was washed with cold methanol, obtaining a dark solid (77 mg, 25%). ^{1}H NMR (400 MHz, $CDCl_3$): δ 10.90 (br s, 2H), 9.52 (br s, 4H), 9.10 (br s,4H), 8.61 (d, J= 8 Hz, 4H), 8.28 (d, J= 8 Hz, 4H), 8.00 (d, J= 4 Hz, 4H), 7.69 (m, 4H), 7.58 (m, 2H). MS (MALDI TOF): m/z 614.2 calcd. for ($C_{44}H_{30}N_4$) 614.7 [M]. UV-vis (CH_2Cl_2): λmax, (nm) 252, 410, 505, 541, 579, 634

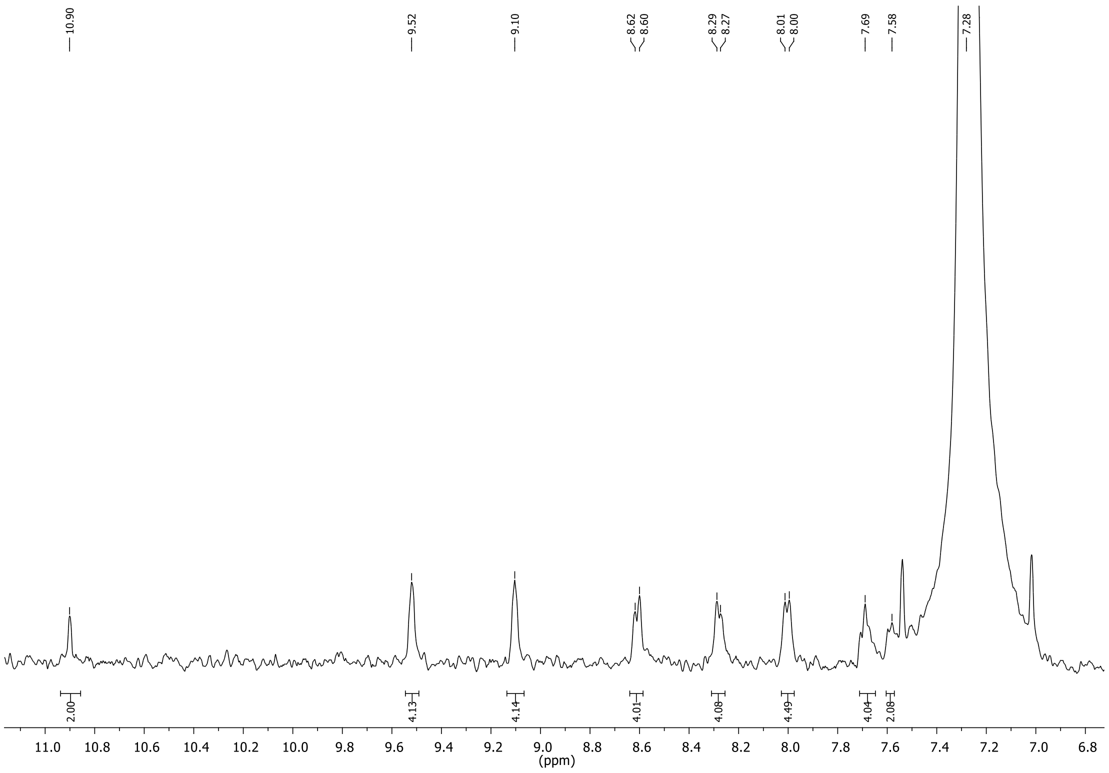

Figure S7.1. ^{1}H-NMR spectrum (400 MHz) in $CDCl_3$ of DBP.

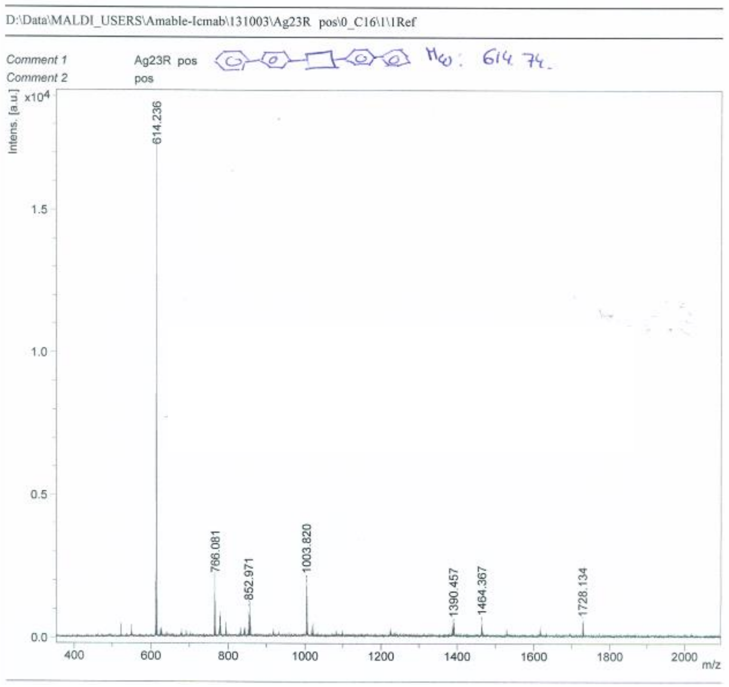

Figure S7.2. MALDI spectrum of DBP.

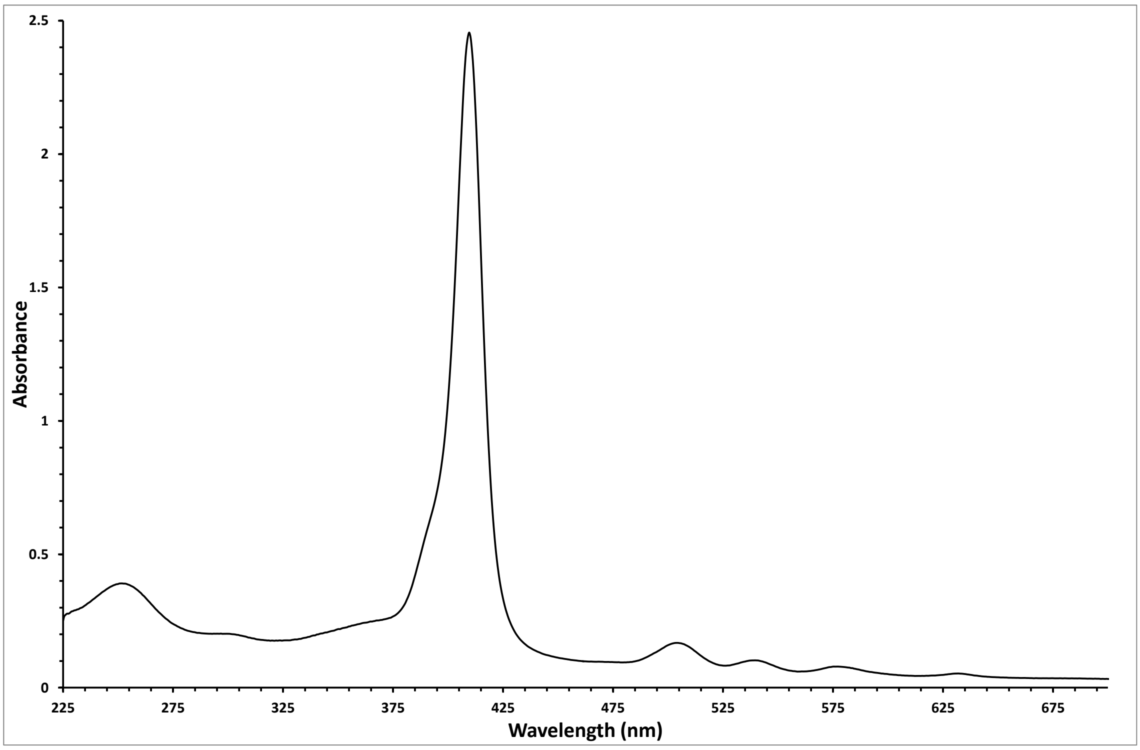

Figure S7.3. UV-Vis spectrum of DBP in CH_2Cl_2.

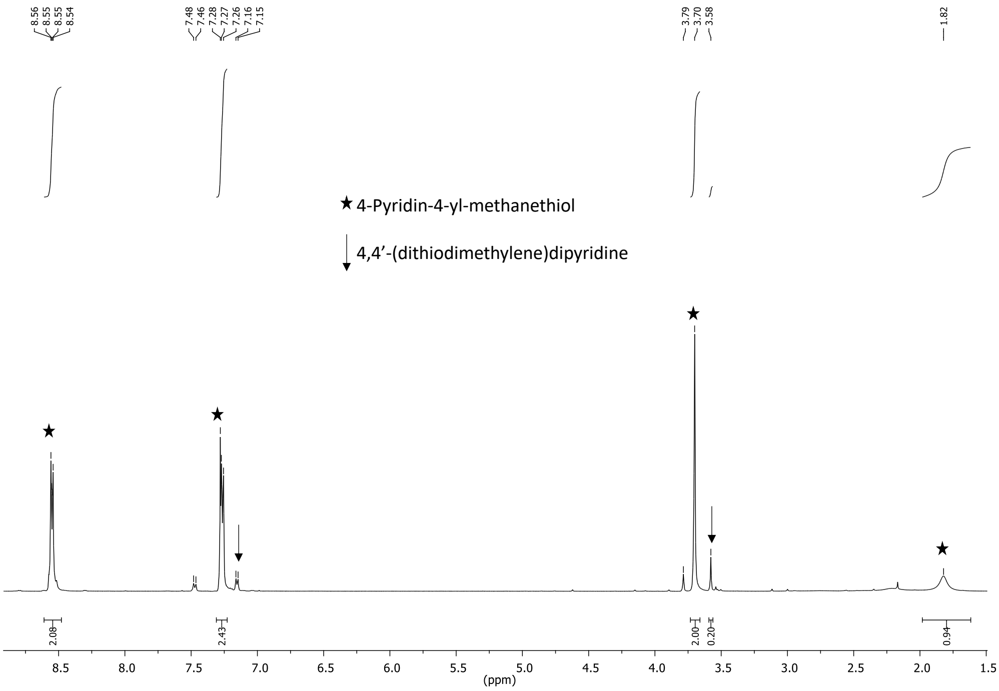

Figure S7.4. ^{1}H-NMR spectrum (360 MHz) in $CDCl_3$ of synthesized pyridin-4-yl-methanethiol.

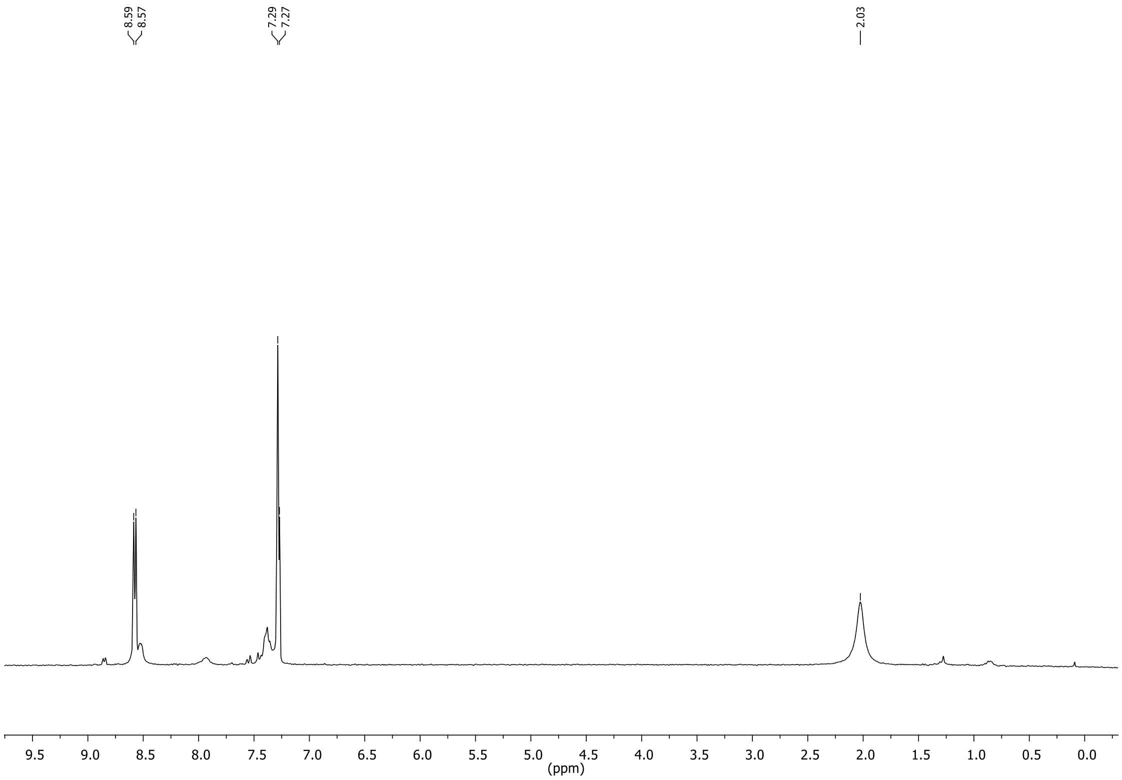

Figure S7.5. ^{1}H-NMR spectrum (250 MHz) in CDCl3 of 4-mercaptopyridine purchased from Sigma-Aldrich

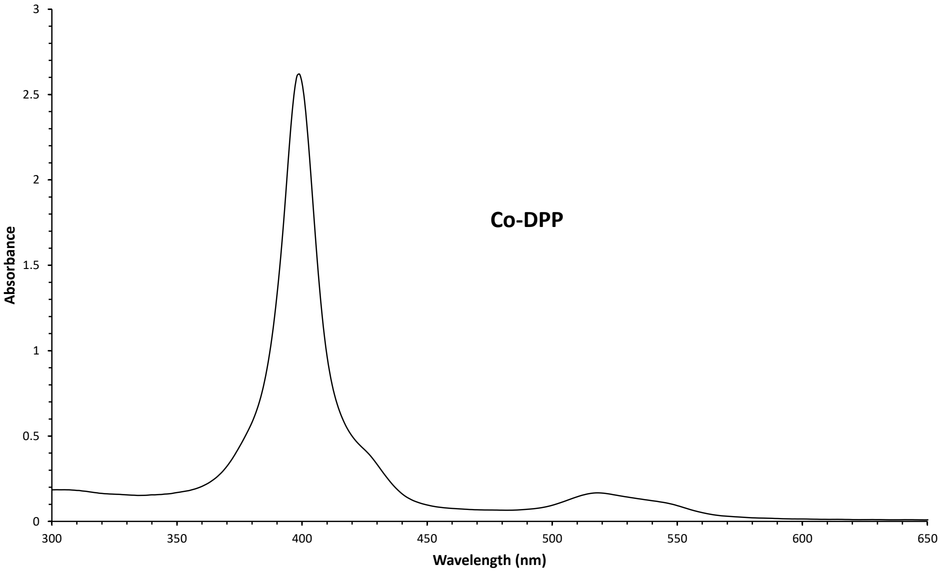

Figure S7.6. UV-Vis spectrum of Co-DPP in CH_2Cl_2.

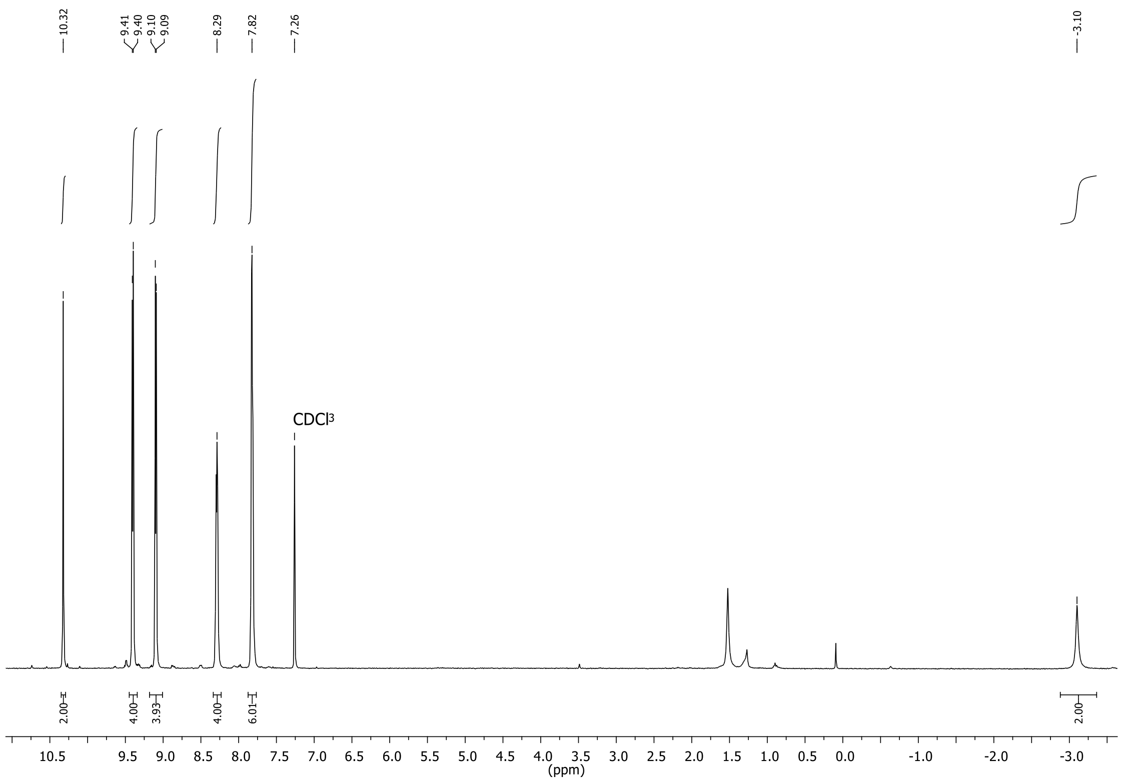

Figure S7.7. ^{1}H-NMR spectrum (250 MHz) in $CDCl_3$ of DPP purchased from Frontier Scientific.

8. References

[1] S. Yoshimoto, M. Yoshida, S. Kobayashi, S. Nozute, T. Miyawaki, Y. Hashimoto, I. Taniguchi, *J. Electroanal. Chem.* **1999**, *473*, 85–92.

[2] A. C. Aragonès, N. Darwish, W. J. Saletra, L. Pérez-García, F. Sanz, J. Puigmartí-Luis, D. B. Amabilino, I. Díez-Pérez, *Nano Lett.* **2014**, *14*, 4751–6.

[3] E. A. Ramírez, E. Cortés, A. A. Rubert, P. Carro, G. Benítez, M. E. Vela, R. C. Salvarezza, *Langmuir* **2012**, *28*, 6839–6847.

[4] S. Stoyanov, I. Petkov, L. Antonov, T. Stoyanova, P. Karagiannidis, P. Aslanidis, *Can. J. Chem.* **1990**, *68*, 1482–1489.

[5] C. Huang, A. V. Rudnev, W. Hong, T. Wandlowski, *Chem. Soc. Rev.* **2015**, *44*, 889–901.

[6] J. M. Soler, E. Artacho, J. D. Gale, A. García, J. Junquera, P. Ordejón, D. Sánchez-Portal, *J. Phys. Condens. Matter* **2002**, *14*, 2745–2779.

[7] J. Ferrer, C. J. Lambert, V. M. García-Suárez, D. Z. Manrique, D. Visontai, L. Oroszlany, R. Rodríguez-Ferradás, I. Grace, S. W. D. Bailey, K. Gillemot, H. Sadeghi, L. A. Algharagholy, *New J. Phys.* **2014**, DOI 10.1088/1367-

2630/16/9/093029.

[8] J. P. Perdew, K. Burke, M. Ernzerhof, *Phys. Rev. Lett.* **1996**, *77*, 3865–3868.

[9] O. A. Vydrov, T. Van Voorhis, *J. Chem. Phys.* **2010**, DOI 10.1063/1.3521275.

[10] J. Hermann, R. A. DiStasio, A. Tkatchenko, *Chem. Rev.* **2017**, *117*, 4714–4758.

[11] C. Toher, S. Sanvito, *Phys. Rev. B* **2008**, *77*, 155402.

[12] X. Ren, P. Rinke, V. Blum, J. Wieferink, A. Tkatchenko, A. Sanfilippo, K. Reuter, M. Scheffler, *New J. Phys.* **2012**, DOI 10.1088/1367-2630/14/5/053020.

[13] V. Blum, R. Gehrke, F. Hanke, P. Havu, V. Havu, X. Ren, K. Reuter, M. Scheffler, *Comput. Phys. Commun.* **2009**, *180*, 2175–2196.

[14] L. Jensen, N. Govind, *J. Phys. Chem. A* **2009**, *113*, 9761–9765.

[15] S. R. Jensen, S. Saha, J. A. Flores-Livas, W. Huhn, V. Blum, S. Goedecker, L. Frediani, *J. Phys. Chem. Lett.* **2017**, DOI 10.1021/acs.jpclett.7b00255.

[16] A. D. Becke, *J. Chem. Phys.* **1993**, DOI 10.1063/1.464913.

[17] T. H. J. Dunning, P. J. Hay, in *Mod. Theor. Chem.* (Ed.: H.F. Schaefer III), Plenum, New York, **1977**, pp. 1–28.

[18] P. J. Hay, W. R. Wadt, *J. Chem. Phys.* **1985**, *82*, 270–283.

[19] W. R. Wadt, P. J. Hay, *J. Chem. Phys.* **1985**, *82*, 284–298.

[20] P. J. Hay, W. R. Wadt, *J. Chem. Phys.* **1985**, DOI 10.1063/1.448975.

[21] B. Xu, N. J. Tao, *Science* **2003**, *301*, 1221–3.

[22] A. C. Aragonès, N. L. Haworth, N. Darwish, S. Ciampi, N. J. Bloomfield, G. G. Wallace, I. Diez-Perez, M. L. Coote, *Nature* **2016**, *531*, 88–91.

[23] Li, J. Hihath, F. Chen, T. Masuda, L. Zang, Tao, *J. Am. Chem. Soc.* **2007**, *129*, 11535–11542.

[24] T. Hines, I. Díez-Pérez, H. Nakamura, T. Shimazaki, Y. Asai, N. Tao, *J. Am. Chem. Soc.* **2013**, *135*, 3319–22.

[25] A. C. A. C. Aragonès, D. Aravena, J. I. J. I. Cerdá, Z. Acís-Castillo, H. Li, J. A. J. A. Real, F. Sanz, J. Hihath, E. Ruiz, I. Díez-Pérez, *Nano Lett.* **2016**, *16*, 218–226.

[26] J. Puigmartí-Luis, W. J. Saletra, A. González, D. B. Amabilino, L. Pérez-García, *Chem. Commun. (Camb).* **2014**, *50*, 82–84.

[27] X. Z. Song, L. Jaquinod, W. Jentzen, D. J. Nurco, S. L. Jia, R. G. Khoury, J. G. Ma, C. J. Medforth, K. M. Smith, J. A. Shelnutt, *Inorg. Chem.* **1998**, *37*, 2009–2019.

[28] Benjamin J. Littler, Mark A. Miller, † Chen-Hsiung Hung, Richard W. Wagner, ‡ Donal F. O'Shea, and Paul D. Boyle, J. S. Lindsey*, **1999**, DOI 10.1021/JO982015+.
